# Targeting miR‐124/Ferroportin signaling ameliorated neuronal cell death through inhibiting apoptosis and ferroptosis in aged intracerebral hemorrhage murine model

**DOI:** 10.1111/acel.13235

**Published:** 2020-10-17

**Authors:** Wen‐Dai Bao, Xiao‐Ting Zhou, Lan‐Ting Zhou, Fudi Wang, Xiaoping Yin, Youming Lu, Ling‐Qiang Zhu, Dan Liu

**Affiliations:** ^1^ Department of Pathophysiology Key Lab of Neurological Disorder of Education Ministry School of Basic Medicine Tongji Medical College Huazhong University of Science and Technology Wuhan China; ^2^ The Institute of Brain Research Collaborative Innovation Center for Brain Science Huazhong University of Science and Technology Wuhan China; ^3^ Department of Nutrition School of Public Health Zhejiang University Hangzhou China; ^4^ Department of Neurology Affiliated Hospital of Jiujiang University Jiujiang China; ^5^ Center for Clinical Precision Medicine Jiujiang University Jiujiang China

**Keywords:** apoptosis, ferroptosis, Fpn, intracerebral hemorrhage, iron, miR‐124

## Abstract

Incidence of intracerebral hemorrhage (ICH) and brain iron accumulation increases with age. Excess iron accumulation in brain tissues post‐ICH induces oxidative stress and neuronal damage. However, the mechanisms underlying iron deregulation in ICH, especially in the aged ICH model have not been well elucidated. Ferroportin1 (Fpn) is the only identified nonheme iron exporter in mammals to date. In our study, we reported that Fpn was significantly upregulated in perihematomal brain tissues of both aged ICH patients and mouse model. Fpn deficiency induced by injecting an adeno‐associated virus (AAV) overexpressing cre recombinase into aged Fpn‐floxed mice significantly worsened the symptoms post‐ICH, including hematoma volume, cell apoptosis, iron accumulation, and neurologic dysfunction. Meanwhile, aged mice pretreated with a virus overexpressing Fpn showed significant improvement of these symptoms. Additionally, based on prediction of website tools, expression level of potential miRNAs in ICH tissues and results of luciferase reporter assays, miR‐124 was identified to regulate Fpn expression post‐ICH. Higher serum miR‐124 levels were correlated with poor neurologic scores of aged ICH patients. Administration of miR‐124 antagomir enhanced Fpn expression and attenuated iron accumulation in aged mice model. Both apoptosis and ferroptosis, but not necroptosis, were regulated by miR‐124/Fpn signaling manipulation. Our study demonstrated the critical role of miR‐124/Fpn signaling in iron metabolism and neuronal death post‐ICH in aged murine model. Thus, Fpn upregulation or miR‐124 inhibition might be promising therapeutic approachs for this disease.

## Graphical abstract

Brain iron accumulation following ICH induced secondary brain injury and neuronal death. However, the mechanisms underlying iron deregulation in aged ICH model is poorly understood. miR‐124/Fpn signaling was downregulated in aged ICH model mice and patients as a protection mechanism. Higher serum miR‐124 levels were correlated with poor neurologic scores of aged patients. Targeting miR‐124/Fpn signaling could reduce the iron accumulation post‐ICH in aged murine model, thus ameliorated hematoma volume, cell apoptosis and neurologic dysfunction through inhibiting apoptosis and ferroptosis.

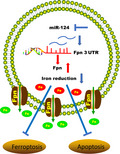

## 1 INTRODUCTION

Spontaneous intracerebral hemorrhage (ICH) has the highest mortality rates among stroke subtypes, and the incidence of intracerebral hemorrhage was increased in elderly patients (Ward, Zucca, Duyn, Crichton, & Zecca, 2014). However, effective therapies are lacking, and translation of the research findings has been limited (Donnan, Hankey, & Davis, 2010). Iron is released from the degradation of the Hb and accumulates in brain tissues around hematomas. During aging, excess iron also accumulates in the substantia nigra, putamen, globus pallidus, caudate nucleus, and cortices, which were associated with motor and cognitive impairment (Ramos et al., 2014; Ward et al., 2014; Zecca, Stroppolo, et al., 2004). Excess iron accumulation leads to oxidative stress, which is detrimental to cell membranes, proteins, and nucleic acids, thereby disrupting cellular function and causing neuronal death (Hentze, Muckenthaler, & Andrews, 2004; Ward et al., 2014; Zecca, Youdim, Riederer, Connor, & Crichton, 2004). The mechanisms of brain iron metabolism are still poorly understood, which greatly limits the development of therapeutic drugs targeting brain iron efflux after ICH. Therefore, elucidating the mechanisms underlying iron metabolism is crucial to developing effective therapeutic strategies to reduce iron accumulation in ICH.

Ferroportin1 (Fpn) is the only mammalian nonheme iron exporter identified to date (Drakesmith, Nemeth, & Ganz, 2015). In the central nervous system, Fpn may distributed in various cell types (Ward et al., 2014), including neurons (Boserup, Lichota, Haile, & Moos, 2011; Duce et al., 2010; Wu et al., 2004), astrocytes (Jeong & David, 2003; Wu et al., 2004), microglia (Urrutia et al., 2013), oligodendrocytes (Boserup et al., 2011), and brain microvascular endothelial cells (Wu et al., 2004). In previous study, the upregulation of hepcidin, which could regulate iron traffic via binding and internalizing Fpn, could induce brain iron accumulation, oxidative injury, and cognitive impairment after ICH (Xiong et al., 2016). However, in this study, they did not show the data of Fpn expression in brain tissues after ICH, and no functional experiments of Fpn were performed. Considering that Fpn expression was upregulated and positively correlated with the ferrous iron status of the brain after ICH (Wang et al., 2015), most likely, the increase of hepcidin could perform its function by reduce the upregulation of Fpn to a lesser degree after ICH. All these results indicated that deregulation of Fpn may play a key role in regulating iron metabolism in ICH. However, the exact roles of Fpn in the pathological process of this disease and the underlying mechanisms were still unclear and need to be further explored.

Understanding the mechanism underlying cell death in response to bleeding in the brain is crucial to developing effective therapeutic strategies. In previous studies, classical apoptotic and necrotic cells have been identified in animal models of ICH (Qureshi et al., 2001), as well as in the perihematoma region after surgical evacuation in humans (Qureshi et al., 2003; Zille et al., 2017). Moreover, recent studies revealed that other forms of regulated nonapoptotic cell death, such as necroptosis and ferroptosis, could also contribute to ICH‐induced cell death (Zille et al., 2017). However, the pathway responsible for iron‐induced cell death remains a mystery.

The goal of this study was to investigate the role and mechanism of pathological deregulation of the iron exporter Fpn in aged ICH model and whether targeting this pathway could reduce neuronal death and improve ICH outcomes. Our study showed that miR‐124 could downregulate Fpn expression in the brain, and both Fpn overexpression and miR‐124 inhibition could alleviate brain edema, iron accumulation, and cell apoptosis in the perihematomal area in aged murine model. Additionally, the serum miR‐124 level was correlated with the clinical outcome of aged patients. We further examined which cell death pathway was associated with miR124/Fpn signaling post‐ICH. The results showed that miR124/Fpn signaling may mediate the outcome of ICH through apoptosis and ferroptosis.

## 2 RESULTS

### 2.1 Fpn was upregulated in perihematomal brain tissues from aged ICH model mice and patients

**Figure 1:**
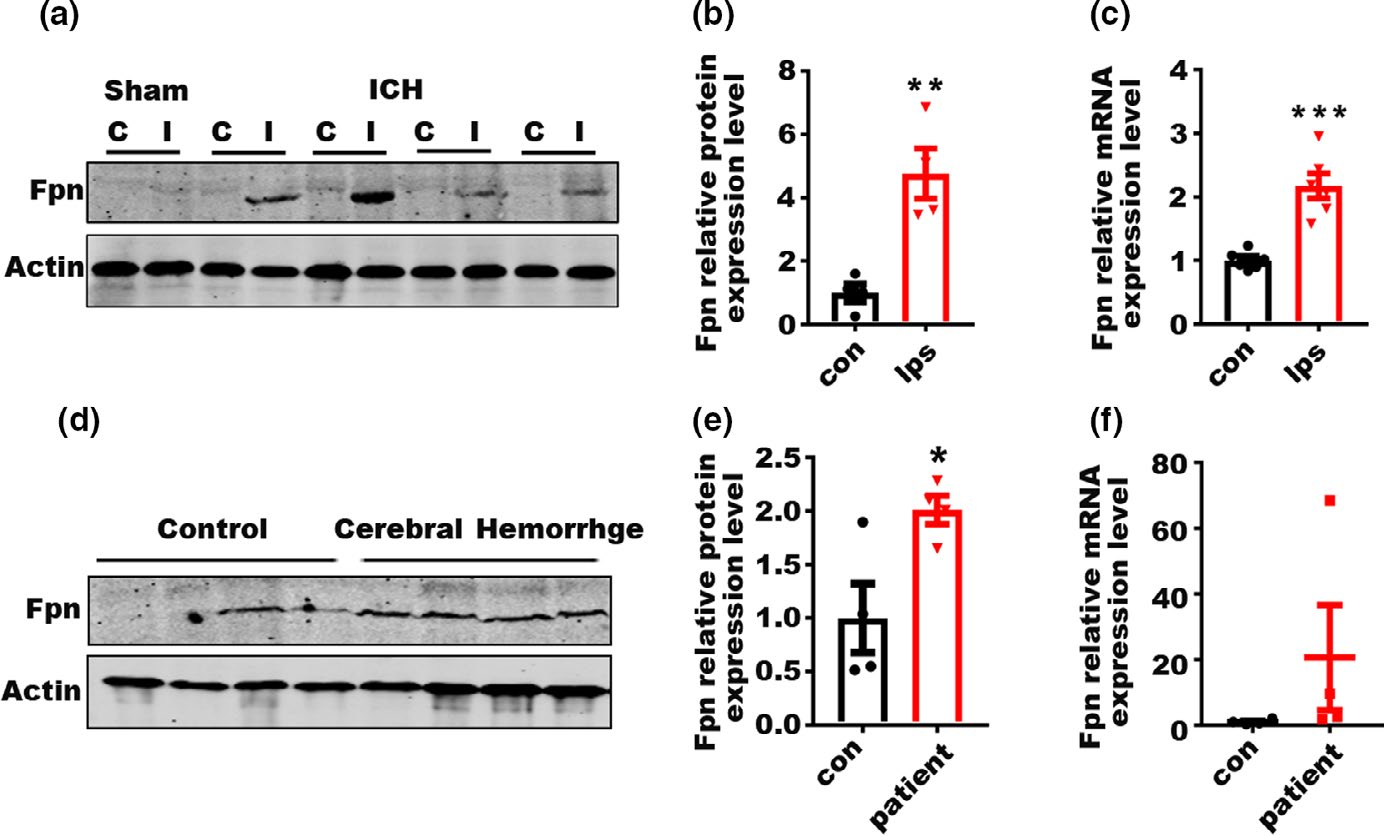
Fpn is upregulated in aged mouse and patient ICH tissues. (a) Protein level of Fpn in the perihematomal brain tissues of the aged ICH mouse model and sham control (20‐month‐old). C, contralateral side; I, ipsilateral side. (b) Quantification for (a), fold change of protein level of Fpn between the contralateral side (con) and ipsilateral side (ips) post‐ICH. (c) mRNA level of Fpn in the tissues of the ICH mouse model. Con, contralateral side; Ips, ipsilateral side (*n* = 6). β‐Actin was used as an internal control, and the results are shown as the fold change of the control. (d) Protein level of Fpn in the perihematomal brain tissues from ICH patients (*n* = 4) and controls (*n* = 4). (e) Quantification of (d). (f) mRNA level of Fpn in the tissues from ICH patients (*n* = 4) and controls (con) (*n* = 4). β‐Actin was used as an internal control, and the results are shown as the fold change of the control. The data are shown as the mean ± *SEM* of at least 3 independent experiments. Statistical analyses were carried out using multiple *t* test. *p < 0.05; **p < 0.01; ***p < 0.001

Brain iron accumulation following ICH‐induced secondary brain injury and neuronal death. Considering that Fpn plays an important role in iron metabolism, we investigated the expression level of Fpn in aged ICH model mice. We observed significant increases in Fpn expression levels in perihematomal brain tissues compared with contralateral tissues and sham control tissues in the mouse model after ICH (Figure 1a–c). The abundance of Fpn mRNA was upregulated 2‐fold (Figure 1c), while the protein level was increased about 5‐fold (Figure 1b). The higher fold change in the protein level indicated the posttranscriptional regulation of Fpn post‐ICH. In perihematomal brain tissues from aged ICH patients (average age above 70), the protein expression levels of Fpn were significantly increased (Figure 1d,e). The mRNA level of Fpn showed no statistical significance between the two groups due to the diversity within the patient group (Figure 1f). These data indicated a dramatic upregulation of Fpn in both the aged ICH mouse model and patients.

### 2.2 Fpn deficiency worsens the symptoms post‐ICH in aged murine model

**Figure 2:**
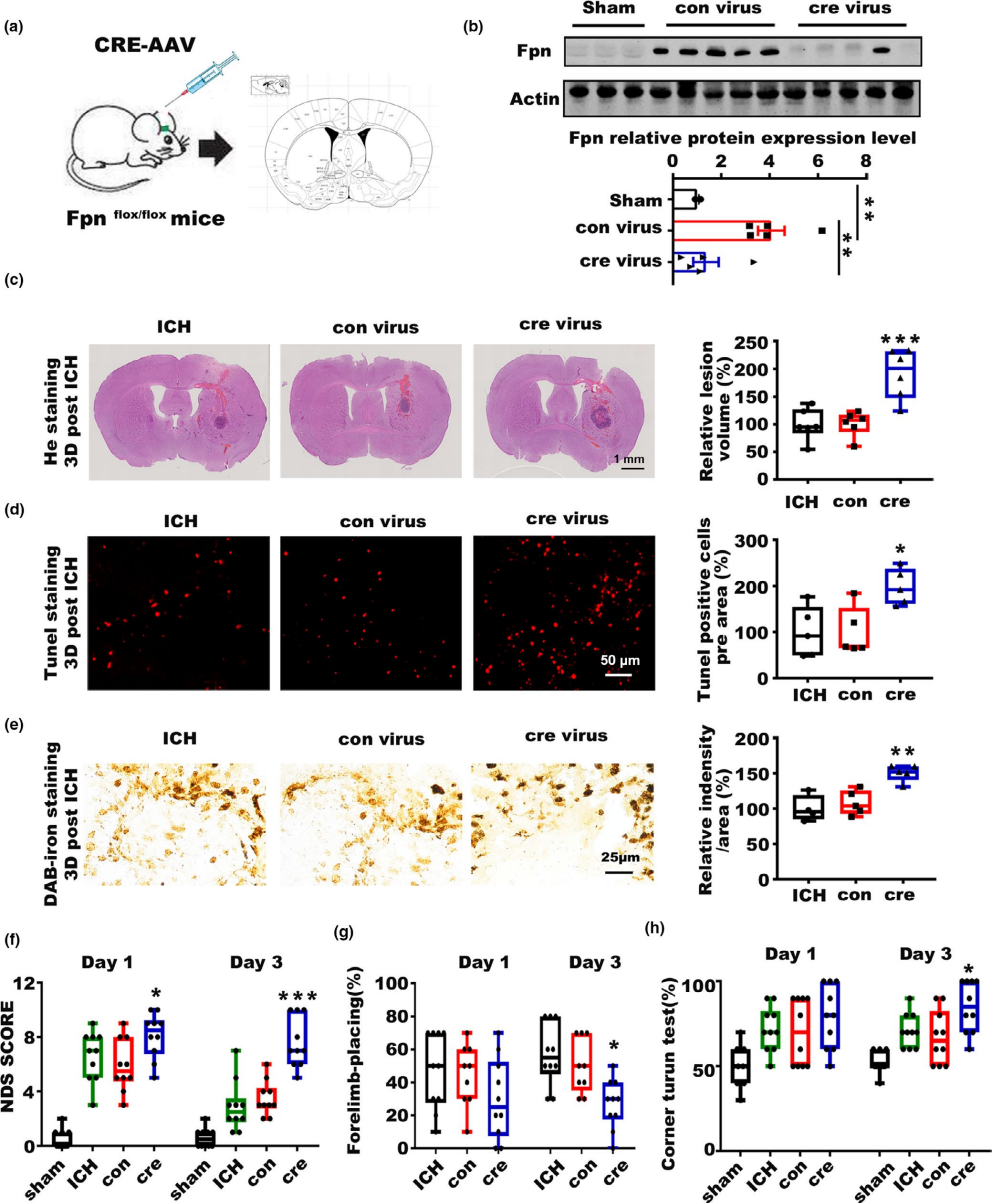
Fpn deficiency worsens the symptoms of ICH. (a) Cartoon outline of the (cre virus) stereotactic injection procedure in aged Fpn‐floxed mice (20‐month‐old). (b) Protein level of Fpn in the brain tissues of the ICH model mice injected with the cre‐expression AAV (cre virus) or control virus (con virus) compared to sham control mice (Sham), the quantitative data were listed below. (c) Brain sections were stained with hematoxylin and eosin (left), and the lesion volume was calculated (right). The results are shown as the fold change (%) of the controls. ICH, mice without any virus injection before the ICH model was generated. Con, mice with con virus injection one month before the ICH model was generated. Cre, mice with cre virus injection one month before the ICH model was generated (*n* = 6). (d) Sections were subjected to TUNEL staining (left), quantification is shown (right), and the results are shown as the fold change (%) of the control (*n* = 5). (e) Perls' staining (left) and quantification of the staining intensity of cells (right) and the results are shown as the fold change (%) of the control. (f) Neurologic deficit score, (g) Forelimb placing capacity, and (h) corner turn test for all these mice post‐ICH (*n* = 9–10, per group). The results are shown as box‐and‐whisker plots (the middle horizontal line within the box represents the median, the boxes extend from the 25th to the 75th percentile, and the whiskers represent 95% confidence intervals). Statistical analyses were carried out using one‐way and two‐way ANOVA. *p < 0.05; **p < 0.01; ***p < 0.001

We then injected an AAV expressing cre recombinase (cre virus) into the corpus striatum of aged *Fpn^flox^* ^/^ *^flox^* mice before ICH. Littermates injected with con‐AAV (con virus) were used as controls (Figure 2a, Figure S1A). The virus injection significantly blocked the induction of Fpn after ICH (Figure 2b), and Fpn deficiency worsened a series of symptoms in the model mice (Figure 2c–h). To evaluate the effects of Fpn deficiency on hematoma volume and iron deposition after ICH, we subjected sections to hematoxylin‐eosin staining (HE) and Perls' blue staining. We stained the brain slices throughout the entire striatum and quantified the hematoma volume of each group. There was a significant increase in the average hematoma volume of the cre virus‐injected group compared with that in the control animals (Figure 2c). Additionally, the cre virus‐injected group had more severe iron deposition than the control group (Figure 2e). Cell apoptosis in the perihematomal area was detected by TUNEL staining, and the number of TUNEL‐positive cells was also increased in the cre virus‐injected group (Figure 2d). NDS (Neurological Deficit Scores) score, forelimb placing, corner turn tests, and rotarod tests were performed at 24 and 72 h after ICH to evaluate the neurologic outcomes. The results showed that cre virus‐injected ICH mice had increased scores on the NDS test (Figure 2f), more severe right forelimb muscle weakness (Figure 2g), and shorter time spent on the rotarod test (Figure S1B). Deficits in correct corner turn preference were also altered by Fpn deficiency (Figure 2h). All of these data clearly showed that Fpn deficiency in ICH worsens brain edema, iron accumulation, and cell apoptosis in the perihematomal area and aggravates the neurologic outcomes. Besides, we also injected AAV‐cre and relative control virus on non‐floxed C57 mice to test the effects of cre recombinase on ICH. The results showed that the overexpression of cre recombinase had no effect on the outcome of ICH in non‐floxed C57 mice (Figure S2).

### 2.3 Fpn overexpression alleviated the symptoms post‐ICH in aged murine model

**Figure 3:**
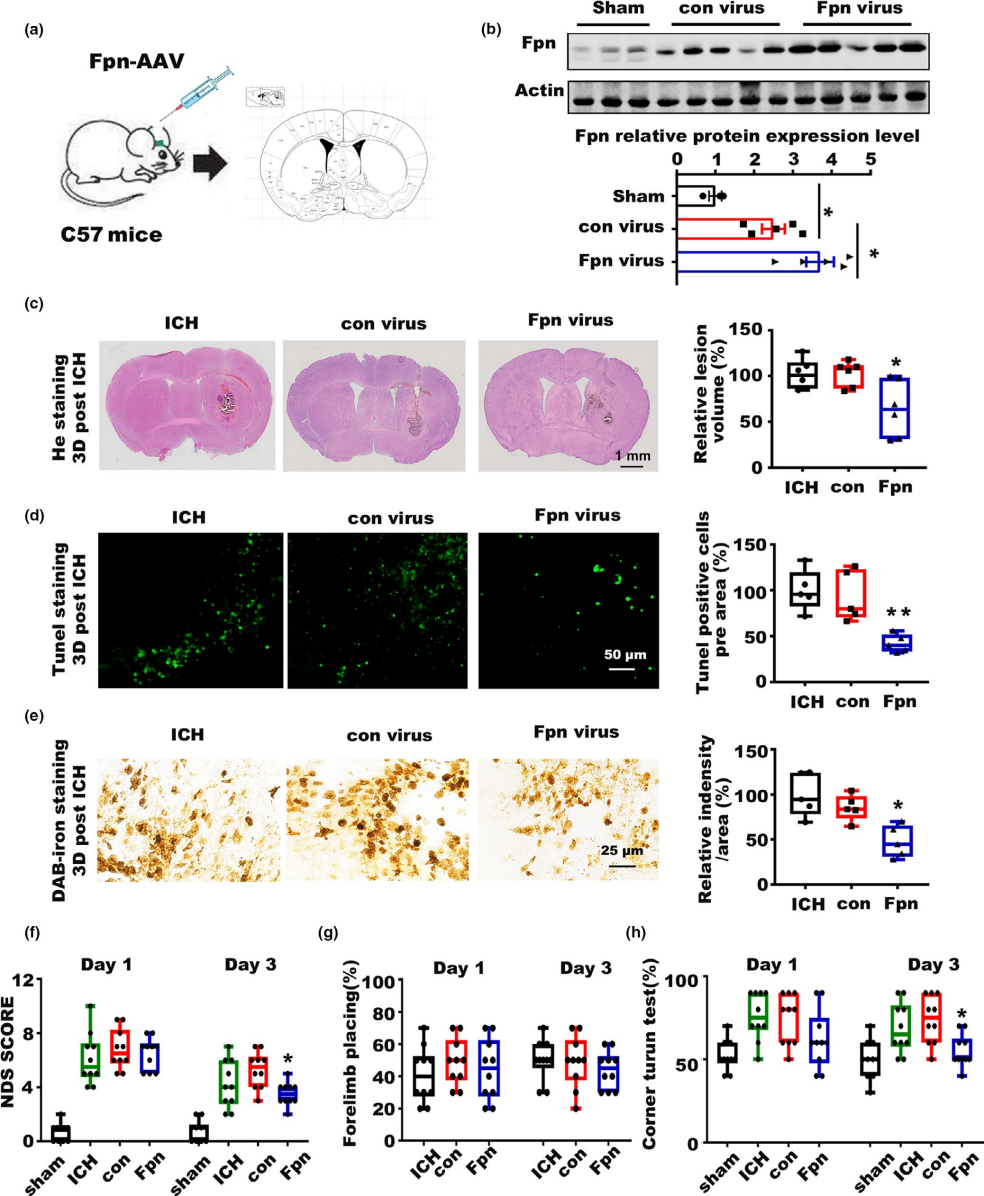
Overexpression of Fpn alleviated the symptoms of ICH. (a) Cartoon outline of the procedure for the stereotactic injection of the AAV overexpressing Fpn in aged C57 mice (20‐month‐old). (b) Protein level of Fpn in the brain tissues of ICH model mice injected with Fpn‐expressing AAV (Fpn virus) or control virus (con virus) compared to that in sham control mice, the quantitative data were listed below. (c) Brain sections were stained with hematoxylin and eosin (left), and lesion volume was calculated (right). The results are shown as the fold change (%) of the controls. ICH, mice without virus injection before the ICH model was generated. Con, mice with con virus injection before the ICH model was generated. Fpn, mice injected with Fpn virus before the ICH model was generated (*n* = 6). (d) Sections were subjected to TUNEL staining (left), and quantification is shown (right). The results are shown as the fold change (%) of the controls (*n* = 5). (e) Perls' staining (left) and quantification of the staining intensity of the cells (right). The results are shown as the fold change (%) of the controls (*n* = 5). (f) Neurologic deficit score, (g) Forelimb placing score, and (h) corner turn test for all the mice after ICH (*n* = 10, per group). Statistical analyses were carried out using one‐way and two‐way ANOVA. *p < 0.05; **p < 0.01; ***p < 0.001

To confirm the protective function of Fpn in ICH, we injected an AAV overexpressing full‐length Fpn into the corpus striatum of aged C57 mice before ICH. C57 mice injected with control AAV were used as controls (Figure 3a, Figure S3A). The virus injection significantly increased Fpn expression after ICH (Figure 3b), and Fpn overexpression alleviated a series of ICH symptoms. There was a significant reduction in the average hematoma volume of the Fpn virus‐injected group compared with the control groups (Figure 3c). Additionally, the Fpn virus‐injected group had less iron deposition than the control group at day 3 (Figure 3e). Cell apoptosis in the perihematomal area was detected by TUNEL staining, and the number of TUNEL‐positive cells was significantly decreased in the Fpn virus‐injected group (Figure 3d). NDS score, forelimb placing and corner turn tests were performed at both 24 and 72 h after ICH to evaluate the neurologic outcomes. The results showed that Fpn virus‐injected ICH mice had decreased scores for the NDS test (Figure 3f), attenuated deficits in the correct corner turn preference and rotarod tests (Figure 3h, Figure S3B). Forelimb muscle weakness was not improved by Fpn overexpression (Figure 3g). The data above clearly showed that Fpn overexpression alleviated brain edema, iron accumulation, and cell apoptosis in the perihematomal area and attenuated the neurologic outcomes post‐ICH.

### 2.4 miR‐124 regulated Fpn expression post‐ICH

**Figure 4:**
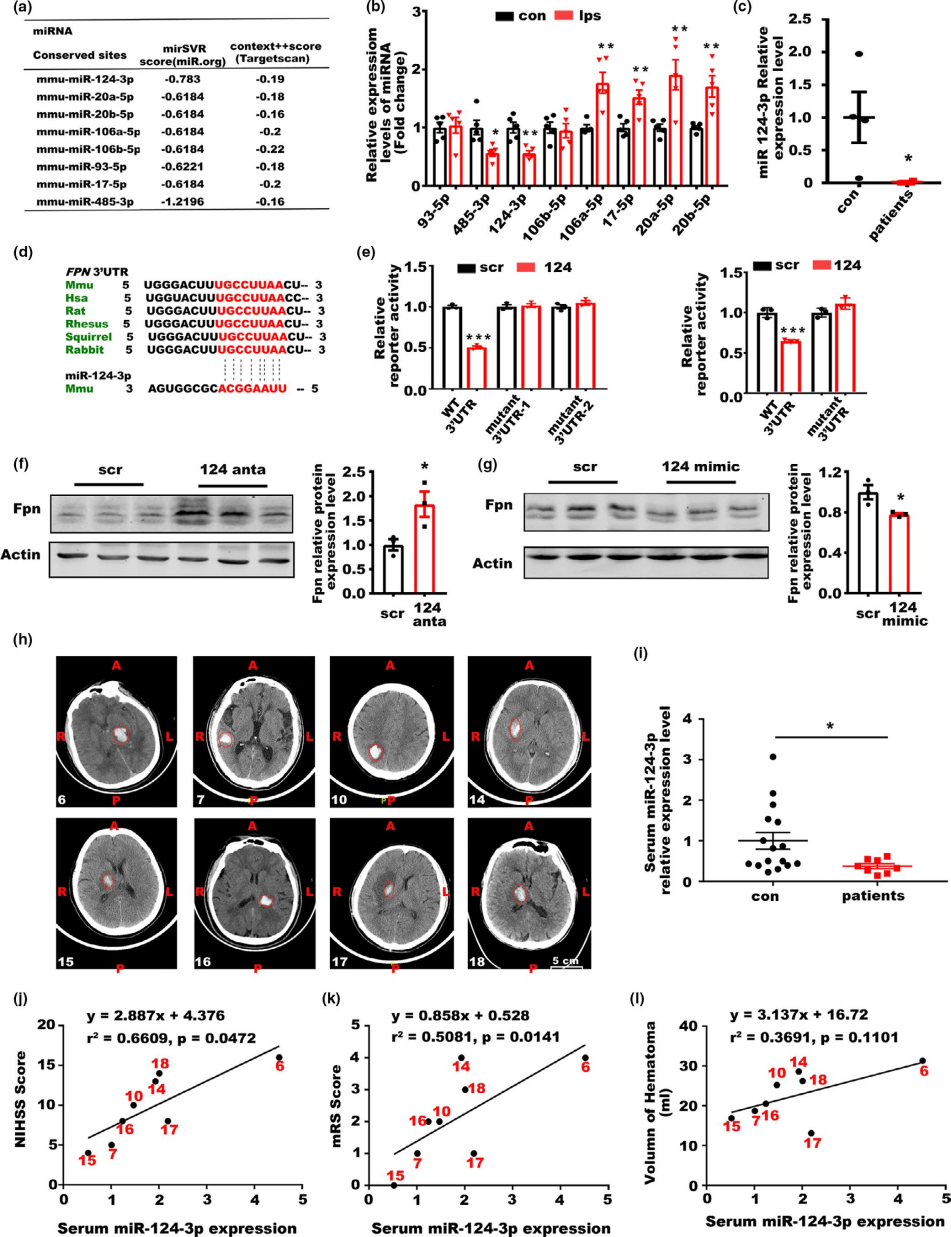
miR‐124 could inhibit Fpn expression post‐ICH. (a) Diagram of the scores of potential microRNAs targeting Fpn predicted by miR.org and Targetscan. (b) Relative level of potential microRNAs in the perihematomal brain tissues of mice post‐ICH compared to the paired contralateral side tissues, the results are shown as the fold change. (c) Expression levels of miR‐124 in the tissues of ICH patients (*n* = 4) and controls (*n* = 4). U6 was used as an internal control, and the results are shown as the fold change of the control. (d) Potential target sites in the 3′UTR of Fpn by miR‐124 across different mammals. (e) 293 T cells were cotransfected with plasmids expressing miR‐124 and reporter plasmids with the WT or mutated (mutant) 3′UTR of mice (left) and human (right) Fpn. Relative luciferase activity was assessed. Paired empty and reporter plasmids expressing the 3′UTR of Fpn were cotransfected as controls, and the results are shown as the fold change of the controls. (f) Protein levels of Fpn in primary neurons transfected with antagomirs and (g) mimics of miR‐124 compared to relative scrambles. (h) MRI pictures of the patients used for analysis, the corresponding serial numbers of the patients are listed in the lower left corner of each picture. (i) Relative level of miR‐124 in the serum from ICH patients (*n* = 8) and control individuals (con) (*n* = 16) (≥65 years old, average age ≥70). The results are shown as the fold change of the control. (j) Correlation analysis between the serum miR‐124 level and the NIHSS score, (k) mRS score (l) and hematoma volume of the patients (*n* = 8). Statistical analyses were carried out using two‐way ANOVA and *t* test. *p < 0.05; **p < 0.01; ***p < 0.001

After we identified the role of Fpn in iron accumulation and neuronal death in ICH, we explored how Fpn expression was regulated here. Interestingly, the fold change in the Fpn protein level was higher than that in the mRNA level in ICH mouse model (Figure 1b,c), suggesting potential posttranscriptional dysregulation for Fpn. In previous studies, hepcidin, which posttranslationally downregulates Fpn, was upregulated after ICH, which is not consistent with the Fpn alterations in this disease, indicating that other mechanisms are involved in Fpn regulation here. To investigate whether microRNAs could target Fpn in the brain, the 3′UTR sequences of both the human and murine Fpn genes were analyzed according to website‐based prediction tools: Targetscan (Lewis, Burge, & Bartel, 2005) (http://www.targetscan.org/vert_72/) and microRNA.org (Betel, Koppal, Agius, Sander, & Leslie, 2010) (http://www.microrna.org/microrna/home.do). Among these microRNAs, we chose 8 candidates with the best scores in both databases and that were conserved across various species (Figure 4a). Then, we examined the expression levels of these candidates in the aged ICH mouse model, the results showed that miR‐485‐3p and miR‐124‐3p were downregulated, while miR‐106a‐5p, miR‐17‐5p, miR‐20a‐5p, miR‐20b‐5p were upregulated after ICH (Figure 4b). The results of luciferase reporter assays showed that miR‐124‐3p, miR‐485‐3p, miR‐106a‐5p could inhibit the activity of the 3′UTR of Fpn in vitro (Figure 4e and Figure S4A). However, only miR‐124‐3p was downregulated in the human samples from ICH patients (Figure 4c, Figure S4B,C). Besides, the basal expression level of miR 485‐3p and miR‐106a‐5p in murine or human brain tissues were also relatively low compared to miR‐124 (Figure 4c, Figure S4D,E).To further determine whether the Fpn 3′UTR is targeted by miR‐124‐3p, we used expression constructs encoding the precursor hairpin sequences to overexpress miR‐124‐3p and measure their respective effects on Fpn 3′UTR reporter activity (Figure 4d,e). Forced miR‐124‐3p expression led to the significant inhibition of both human and mouse Fpn 3′UTR reporter activity, while the Fpn 3′UTR reporter plasmids with mutant sequences of the miR‐124 binding sites were not inhibited by miR‐124‐3p overexpression (Figure 4e). We further confirmed the regulation of Fpn by miR‐124‐3p in primary neurons, antagomirs of miR‐124 increased Fpn expression by inhibition of miR‐124, while mimics of miR‐124 inhibited Fpn expression (Figure 4f,g). All these results indicated that miR‐124‐3p may be attributed to the increase of Fpn in ICH.

### 2.5 Serum miR‐124 levels were correlated with the clinical outcomes of elder ICH patients

To further explore the clinical significance of miR‐124 in ICH, we analyzed the expression of miR‐124 in the serum of ICH patients over 65 years old and age‐matched controls using microRNA qPCR panels (Figure 4h,i). miR‐124 levels were decreased in the serum samples of ICH patients compared with those of the controls (Figure 4i). Additionally, correlations between miR‐124 serum levels and NIHSS (National Institute of Health stroke scale) score (Figure 4j), mRS (Modified Rankin Scale) score (Figure 4k), hematoma volume (Figure 4l), and gender (Figure S5) were determined. The analysis of serum miR‐124 and clinicopathologic parameters revealed that high serum miR‐124 levels were associated with more serious NIHSS scores and mRS scores but not with hematoma volume, which indicated a more complex status in human patients than in the murine model. The serum miR‐124 level was not affected by gender (Figure S5). In conclusion, high serum miR‐124 levels were associated with poor clinicopathologic parameters and neurological scores in aged ICH patients.

### 2.6 Inhibition of miR‐124 alleviated the symptoms post‐ICH in aged murine model

**Figure 5:**
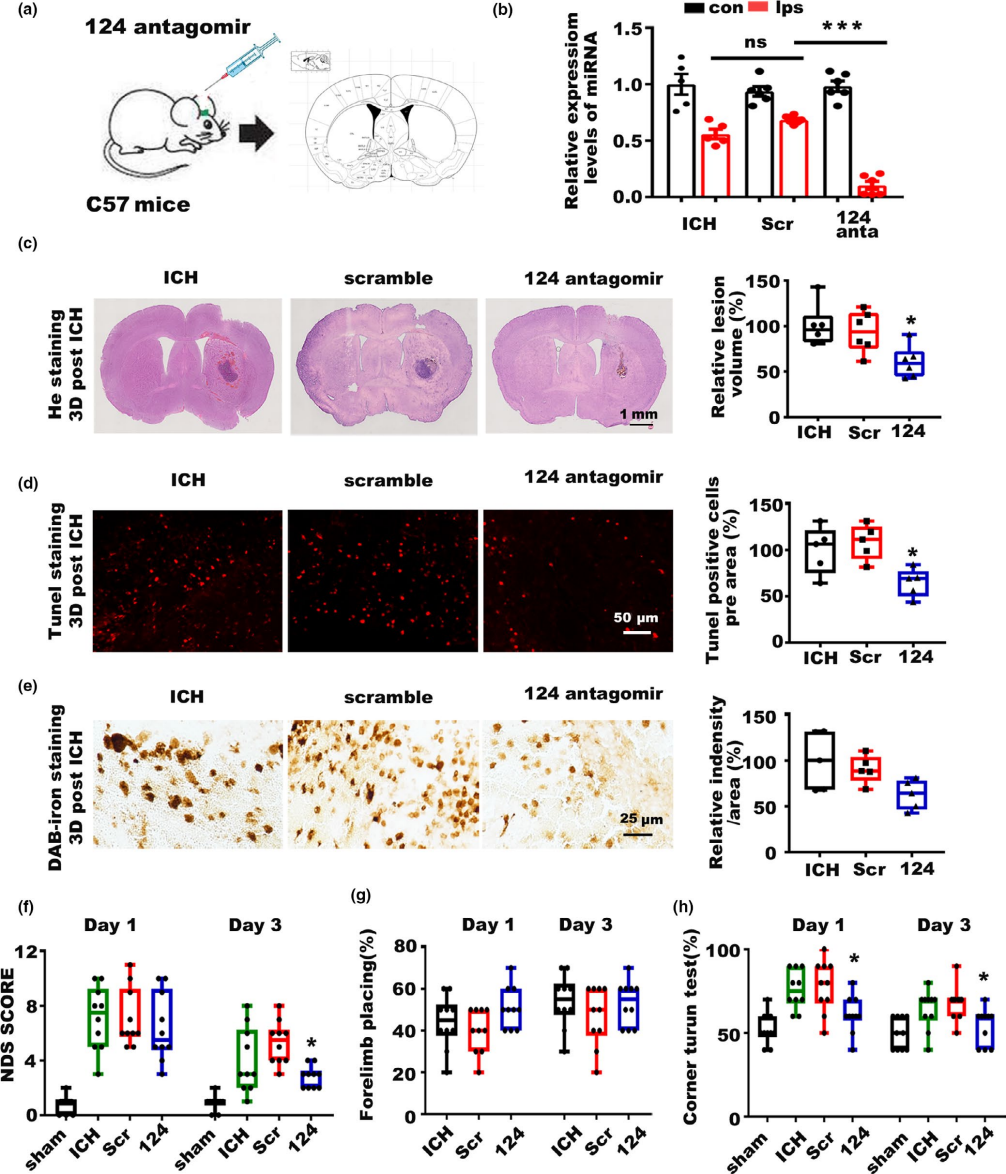
Inhibition of miR‐124 alleviated the symptoms of ICH. (a) Cartoon outline of the stereotactic injection of the antagomir targeting miR‐124 in aged C57 mice (20‐month‐old). (b) miR‐124 levels in the brain tissues of ICH model mice between the contralateral side (con) and ipsilateral side (ips) injected with miR‐124 antagomir (124 anta), scrambled control (Scr), and blank control (ICH). (c) Brain sections were stained with hematoxylin and eosin (left), and lesion volume was calculated (right). The results are shown as the fold change (%) of the controls. ICH, mice without injection before ICH induction. Scr, mice with scrambled antagomir injection before ICH induction. 124, mice with miR‐124 antagomir injection before ICH induction (*n* = 6). (d) Sections were subjected to TUNEL staining (left), and quantification is shown (right). The results are shown as the fold change (%) of the controls (*n* = 5). (e) Perls' staining (left) and quantification of the staining intensity of the cells (right). The results are shown as the fold change (%) of the controls (*n* = 5). (f) Neurologic deficit score, (g) Forelimb placing score, and (h) corner turn test for all these mice after ICH (*n* = 10, per group). Statistical analyses were carried out using one‐way and two‐way ANOVA. *p < 0.05; **p < 0.01; ***p < 0.001

**Figure 6:**
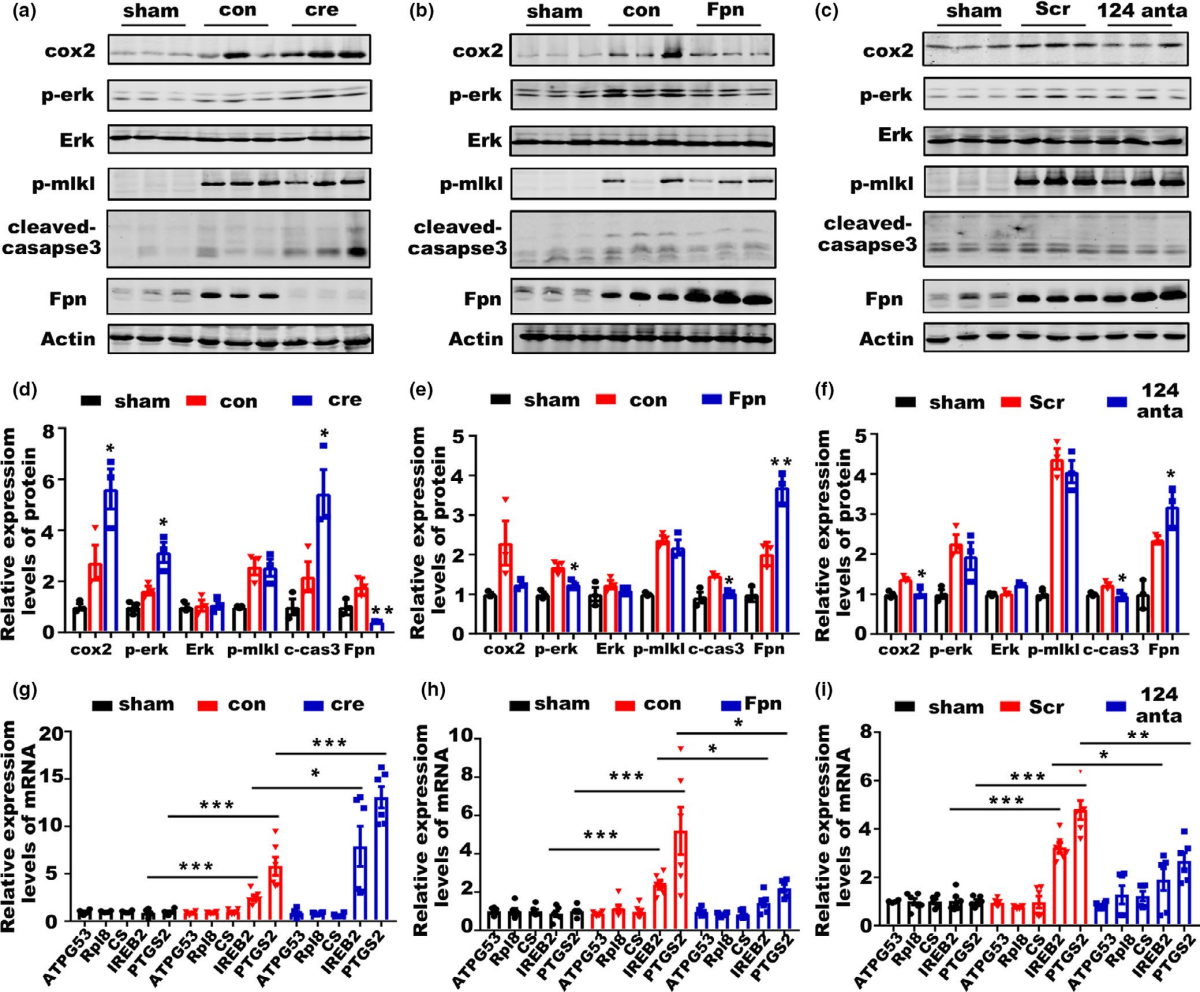
miR‐124/Fpn signaling mediated the outcome of ICH through apoptosis and ferroptosis. (a) Protein levels of cox2, p‐Erk 1/2, Erk 1/2, p‐mlkl, cleaved‐caspase3, and Fpn in the perihematomal brain tissues of post‐ICH mice preinjected with cre‐AAV (cre) or con virus (con) compared to sham control mice (sham). (b) Protein levels of cox2, p‐Erk 1/2, Erk 1/2, p‐mlkl, cleaved‐caspase3, and Fpn in the perihematomal brain tissues of post‐ICH mice preinjected with fpn‐AAV (Fpn) or con virus (con) compared to sham control mice (sham). (c) Protein levels of cox2, p‐Erk 1/2, Erk 1/2, p‐mlkl, cleaved‐caspase3, and Fpn in the perihematomal brain tissues of post‐ICH mice preinjected with miR‐124 antagomir (124) or scrambled control (con) compared to sham control mice (sham). (d) The quantification for the protein levels of cox2, p‐Erk 1/2, Erk 1/2, p‐mlkl, cleaved‐caspase3, and Fpn in the perihematomal brain tissues of mice after ICH in figure a (*n* = 3) (e) figure b (*n* = 3), (f) and figure c (*n* = 3). (g) Relative mRNA levels of ATPG53, Rpl8, CS, IREB2, and PTGS2 in the brain tissues of post‐ICH mice preinjected with cre‐AAV (cre) or con virus (con) compared to those of sham control mice (sham), (h) in the brain tissues of post‐ICH mice preinjected with fpn‐AAV (Fpn) or con virus (con) compared to sham control mice (sham), (i) and in the brain tissues of post‐ICH mice preinjected with miR‐124 antagomir (124) or scrambled control (con) compared to sham control mice (sham) (*n* = 6). Statistical analyses were carried out using two‐way ANOVA and multiple *t* test. *p < 0.05; **p < 0.01; ***p < 0.001

To confirm the regulation of Fpn by miR‐124 in ICH, we injected an antagomir that could effectively reduce the miR‐124 level, into the corpus striatum of aged C57 mice before ICH. C57 mice injected with scrambled antagomirs were used as control groups (Figure 5a). The antagomir injection significantly reduced the miR‐124 level (Figure 5b) and upregulated Fpn post‐ICH (Figure 6c,f), which alleviated a series of ICH symptoms. There was a significant reduction in the average hematoma volume of the miR‐124 antagomir‐injected group compared with the control group (Figure 5c). The number of TUNEL‐positive cells was also decreased in the miR‐124 antagomir‐injected group (Figure 5d), indicating a reduction in cell apoptosis. Though the average value of tissue iron content of the perihematomal region in antagomir‐injected group was lower than the control group; however, the difference had no statistical significance due to the diversity within groups (Figure 5e). The results of behavioral tests showed that antagomir‐injected ICH mice had decreased NDS test scores (Figure 5f), and attenuated deficits in the correct corner turn preference tests (Figure 5h). Forelimb muscle weakness was not improved by miR‐124 inhibition (Figure 5g). Besides, we test the effect of antagomir‐124 in Fpn knockout animals (Fpn‐floxed animals injected with AAV‐Cre) to demonstrate the function of regulation of Fpn by miR‐124 in ICH, and the results showed that Fpn deficiency abolished the alleviation of the symptoms of ICH by antagomir of miR‐124 (Figure S6). Thus, we identified the importance of miR‐124/Fpn signaling in the regulation of iron accumulation and cell death, and suppression of this signaling effectively alleviated the symptoms of ICH.

### 2.7 miR‐124/Fpn signaling probably mediated the neuronal death post‐ICH through apoptosis and ferroptosis

Various forms of cell death have been identified after ICH. To evaluate which form was responsible for miR‐124/Fpn signaling‐mediated cell death in ICH, we examined the molecular characteristics of apoptosis, necroptosis, and ferroptosis after manipulating the miR‐124/Fpn signaling. The protein level of cleaved‐caspase 3 was significantly increased by Fpn deficiency after ICH (Figure 6a,d), implying increased cell apoptosis under this condition, which was consistent with the TUNEL staining results (Figure 2d). Moreover, Fpn overexpression or miR‐124 inhibition could effectively reduce the protein levels of cleaved caspase3 and cell apoptosis signals (Figures 6b,c,e,f, 3d and 5d,). Additionally, ferroptosis‐related genes were also affected by Fpn expression. Both cox2 and p‐erk were induced by Fpn knockout in ICH model, and Fpn overexpression could rescue this upregulation of cox2 and p‐erk; in contrast, inhibition of miR‐124 affected cox2 but not p‐erk (Figure 6a‐f). To further investigate whether ferroptosis‐related gene sets are regulated by Fpn expression, we examined the mRNA expression of ATP5G3, RPL8, CS, and IREB2(Dixon et al., 2012), as well as PTGS2, which has been shown to be induced during ferroptosis (Yang et al., 2014). Consistent with previous studies, PTGS2 and IREB2 mRNA levels were significantly increased after ICH (Chang, Cho, & Wang, 2014). The increased PTGS2 and IREB2 expression could be attenuated by Fpn overexpression or miR‐124 inhibition (Figure 6g‐i). Necroptosis does not appear to be involved in miR‐124/Fpn signaling‐mediated cell death in ICH. P‐MLKL was dramatically upregulated by ICH, which could not be ameliorated by Fpn or miR‐124 manipulation (Figure 6a–c). Together, these findings suggest that miR‐124/Fpn signaling mediates cell death during ICH through apoptosis and ferroptosis but not necroptosis.

## 3 DISCUSSION

The release of iron from hemoglobin degradation products after red blood cell hemolysis induces iron accumulation within the perihematomal region. Excess iron has been implicated in various processes that contribute to neuronal injury after ICH, including reactive oxygen species (ROS) production, lipid peroxidation, inflammation, and autophagy (Chen et al., 2012; Li et al., 2017; Wu, Wu, Xu, Wang, & Wang, 2011). During aging, different iron complexes also accumulate in brain. However, the mechanisms of brain iron metabolism underlying the pathological process remain elusive, which greatly limits the development of therapeutic drugs targeting brain iron accumulation after ICH. Previous studies provide evidence of the effects of increased hepcidin on aggravating oxidative brain injury and cognition impairment in ICH mice (Xiong et al., 2016). Hepcidin has been shown to regulate cellular iron efflux by binding to Fpn and inducing its internalization in neuronal cells (Nemeth et al., 2004). Nevertheless, the function and regulation of Fpn after ICH have not been well examined. To study the role of Fpn in different mouse tissues, the Fpn locus was targeted, and loxP sites were introduced into introns flanking exons 6 and 7 to generate a “floxed” allele (Donovan et al., 2005). A series of studies were performed based on this mouse model to show the function of Fpn1 in hepatocytes, macrophages, and red blood cells (Zhang et al., 2011, 2012, 2018). In this study, we knocked out Fpn in the corpus striatum by stereotactic injection of cre virus before ICH. Fpn deficiency in the perihematomal region significantly exacerbated iron accumulation, cell apoptosis, and behavior test impairments after ICH, which indicated the important role of Fpn in iron metabolism and iron‐induced neuronal death in vivo.

The regulation of Fpn expression in different physical conditions is complicated. In previous studies, Fpn was shown to be regulated at multiple levels—transcriptionally by heme (Marro et al., 2010), posttranscriptionally by the IRP system (Hentze et al., 2004), and posttranslationally by the iron regulatory hormone hepcidin (Drakesmith et al., 2015; Nemeth et al., 2004). In addition, Fpn expression could also be posttranscriptionally regulated by miR‐485‐3p, which is induced during iron deficiency and represses Fpn expression by directly targeting the Fpn 3′UTR (Sangokoya, Doss, & Chi, 2013). However, no studies to date have sought to identify the potential repertoire of microRNAs that could regulate iron accumulation or target the iron exporter Fpn in the brain. Eight candidate microRNAs were chosen according to predictions of unbiased website database tools. Among all these candidates, only miR‐124‐3p was downregulated in both the mouse model and brain tissues from ICH patients, which was consistent with Fpn upregulation in the perihematomal region. The luciferase reporter assay results confirmed the regulation of the Fpn 3′UTR in vitro. Forced miR‐124‐3p overexpression in primary neurons resulted in Fpn expression inhibition, while miR‐124‐3p inhibition in the mouse brain could upregulate Fpn expression in vivo. Collectively, these data clearly support a role for miR‐124‐3p as an important posttranscriptional regulator of endogenous Fpn expression in brain tissues after ICH.

The research of the expression and function of miR‐124‐3p after ICH in the brain is still elusive. In our study, miR‐124 levels in the perihematomal brain tissue of aged ICH mouse model and human patients were measured by real‐time PCR. We found that miR‐124 levels in perihematoma tissue were lower in ICH model mice than in sham mice, which was consistent with a previous study (Yu et al., 2017). In their study, they demonstrated neuroprotective effect of miR‐124 in ICH model which were generated in young C57BL/6 mice (8–10 weeks), while in our study aged Fpn‐floxed mice and aged C57 (20‐month‐old) were used in the mice model. Considering the diversified roles of miR‐124 in aging‐related neurodegenerative disease (Caldeira et al., 2017; Kong, Wu, Zhang, Wan, & Yuan, 2015; Wang et al., 2018), the different results between these two studies probably due to the age of model mice, which inferred that the therapy designed for ICH patients should consider individual difference especially the age. We also detected miR‐124 levels in serum and brain tissues from ICH patients, and these results agreed with the mouse model. Furthermore, the levels of miR‐124 in the serum from ICH patients were positively correlated with the NIHSS score and mRS score, which indicated the important role of miR‐124 in the development and behavior deficits after ICH. However, in another study, miR‐124 expression was found to be increased in rat ICH and human plasma samples (Wang et al., 2018). In our study, we specifically address the expression of miR‐124 in aged murine model post‐ICH. Besides, the patient samples used in this study were all elder patients ≥65 years old, and the average age of these patients was above 70 years old. Thus, the inconsistent results regarding the levels of serum miR‐124 may be due to different experimental models and the age of mice and patients that may influence miR‐124 expression in brain tissue.

Various forms of cell death have been identified after ICH, including apoptosis (Matsushita et al., 2000), necrosis (Zhu et al., 2012), and ferroptosis (Li et al., 2017; Zille et al., 2017) in humans and experimental animals. In previous studies, inhibition of apoptosis, necrosis, and ferroptosis could improve outcomes in animals subjected to experimental ICH (Grossetete & Rosenberg, 2008; Zhu et al., 2012; Zille et al., 2017). However, no successful clinical trials using any single cell death inhibitor have been reported. In our study, we examined the expression of cell death‐related genes after manipulating Fpn or miR‐124 levels to identify which type of cell death was involved in miR‐124/Fpn signaling‐mediated cell death. Either Fpn overexpression or miR‐124 inhibition could effectively alleviate apoptosis after ICH as evidenced by decreased TUNEL staining and reduced cleavage of caspase3. In addition, the expression of ferroptosis‐related genes was also altered after manipulating miR‐124/Fpn signaling in ICH, while necrosis seemed to be not involved in this signaling‐induced neuronal death.

In the present study, we demonstrated the crucial role of miR‐124/Fpn signaling in iron accumulation and neuronal death after ICH. We found the following results: (a) The expression level of Fpn was increased post‐ICH in aged murine model and patients. (b) Fpn overexpression could reduce iron accumulation and cell apoptosis in the perihematomal area and attenuated the neurologic outcomes in experimental ICH, while Fpn knockout worsened all of these phenotypes. (c) miR‐124 regulated Fpn expression in ICH, and the level of miR‐124 in serum was positively correlated with the neurologic scores of the patients. (d) miR‐124/Fpn signaling‐mediated neuronal cell death after ICH through apoptosis and ferroptosis, but not necroptosis. Taken together, our findings demonstrate that miR‐124/Fpn signaling plays a key role in regulating iron overload and neuronal cell death after ICH. Overexpression of Fpn or inhibition of miR‐124 attenuates the symptoms of ICH, which provides a promising therapeutic strategy for treating this condition.

## 4 EXPERIMENTAL PROCEDURES

### 4.1 Mice

Fpn‐floxed (*Fpn^flox^* ^/^ *^flox^*) mice (Zhang et al., 2012) were obtained from Dr. N.C. Andrews and transferred into a C57bl/6 background. Genotyping was performed as previously described (Donovan et al., 2005; Goebbels et al., 2006). Ninety aged Fpn‐floxed mice and one hundred twenty aged C57bl/6 (20‐month‐old) were used in this study. All of the mice were maintained in specific pathogen‐free husbandry and fed a standard rodent diet. All the mice were bred in the Experimental Animal Central of Tongji Medical College, Huazhong University of Science and Technology. This study was approved by Animal Care and Use Committee of Tongji Medical College.

### 4.2 Human Brain Samples collection

Elderly patients with acute cerebral hemorrhage were registered at the Second Affiliated Hospital of Nanchang University as previously described (Ouyang et al., 2019). Eligible patients were ≥65 years old and the average age of this patient was above 70 years old. Patient serum samples (*n* = 8), control serum samples (*n* = 16), patient perihematoma brain tissues samples (*n* = 4), and control brain tissue samples (*n* = 4) were obtained after admission. The Medical Ethics Committee of the Second Affiliated Hospital of Nanchang University, Nanchang, China, approved all patient blood sample and tissue sample collections.

### 4.3 Immunofluorescence and immunohistochemistry

Details were described in Appendix S1 (Supplementary Experimental Procedures).

### 4.4 Virus and stereotaxic injection

Details were described in Appendix S1 (Supplementary Experimental Procedures).

### 4.5 ICH models

The detailed procedures used to construct the ICH model were established in previous studies (Rynkowski et al., 2008; Xiong et al., 2016). Briefly, mice were treated with a mixture of ketamine (100 mg/kg) and dexmedetomidine (0.5 mg/kg) and immobilized on a stereotaxic apparatus (RWD Life Science Co., Shenzhen). All experimental mice received a total of 20 μl autologous blood injected successively into the caudate nucleus (bregma 0: 0.8 mm anterior, 2 mm left lateral and 3.5 mm deep). Unsuccessful ICH models, including asymptomatic and dead mice before sacrifice, were excluded from this study.

### 4.6 Neurological deficit scores and behavioral tests

Three behavioral tests, the forelimb placing test, the corner turn test, and the rotarod test, were used to quantify sensorimotor function at 24 and 72 h after hemorrhage. The detailed procedures were established in previous studies (Hua et al., 2002; Rynkowski et al., 2008; Shiotsuki et al., 2010; Zhang et al., 2002). Details were described in Appendix S1 (Supplementary Experimental Procedures).

### 4.7 Luciferase activity assay

For miR‐124 test, 293 T cells were cotransfected with the pcDNA‐R‐124P‐HA plasmid, which overexpressed miR‐124 or pcDNA‐scrambled‐HA as a control vector and wild‐type or mutant Fpn 3′UTR plasmid. Both the murine and human Fpn 3′UTR plasmid were used for test. For other miRNAs test, 293 T cells were cotransfected with relative mimics or scrambled control and wild‐type or mutant Fpn 3′UTR plasmid. Details of Luciferase Activity Assay were described in Appendix S1 (Supplementary Experimental Procedures).

### 4.8 Western blot analysis and quantitative RT‐PCR

Details of Western blot analysis and quantitative RT‐PCR were described in Appendix S1 (Supplementary Experimental Procedures).

### 4.9 Statistical analysis

GraphPad Prism was used for statistical analysis. Univariate and multivariate linear regression were used to evaluate correlations between continuous variables. The data are presented as the mean ± *SEM*. Unpaired *t* tests (two‐tailed) were used for single comparisons, and one‐way or two‐way ANOVAs were used for multiple comparisons. A *p* value <0.05 was considered significant.

## CONFLICT OF INTEREST

The authors have declared that no conflict of interest exists.

## AUTHOR CONTRIBUTIONS

Ling‐Qiang Zhu and Dan Liu conceived this study. Wen‐Dai Bao, Xiao‐Ting Zhou performed the experiments. Wen‐Dai Bao and Ling‐Qiang Zhu analyzed the data. Ling‐Qiang Zhu, Dan Liu, Wen‐Dai Bao designed and coordinated the investigations. Fudi Wang, Youming Lu participate the discussion and provide experimental guide. Ling‐Qiang Zhu and Wen‐Dai Bao wrote the manuscript. Xiaoping Yin dealt with the human samples. The final version of the manuscript was approved by all authors.

## Data Availability

The data that support the findings of this study are available from the corresponding author upon reasonable request.
