# Correction to “Targeting miR‐124/ Ferroportin Signaling Ameliorated Neuronal Cell Death Through Inhibiting Apoptosis and Ferroptosis in Aged Intracerebral Hemorrhage Murine Model”

**DOI:** 10.1111/acel.70006

**Published:** 2025-02-13

Bao, W.‐D., X.‐T. Zhou, L.‐T. Zhou, et al. 2020. “Targeting miR‐124/Ferroportin Signaling Ameliorated Neuronal Cell Death Through Inhibiting Apoptosis and Ferroptosis in Aged Intracerebral Hemorrhage Murine Model.” *Aging Cell* 19: e13235. https://doi.org/10.1111/acel.13235.

During the data organization of this manuscript, there was an error inadvertently incorporated into the manuscript. We mistakenly placed a duplicated image of iron staining for two control groups: the ICH and con virus group in Figure 2E. All other parts of this article remain intact, valid, and unchanged.

The corrected version of Figure 2E now contains correct images, and it is shown below:

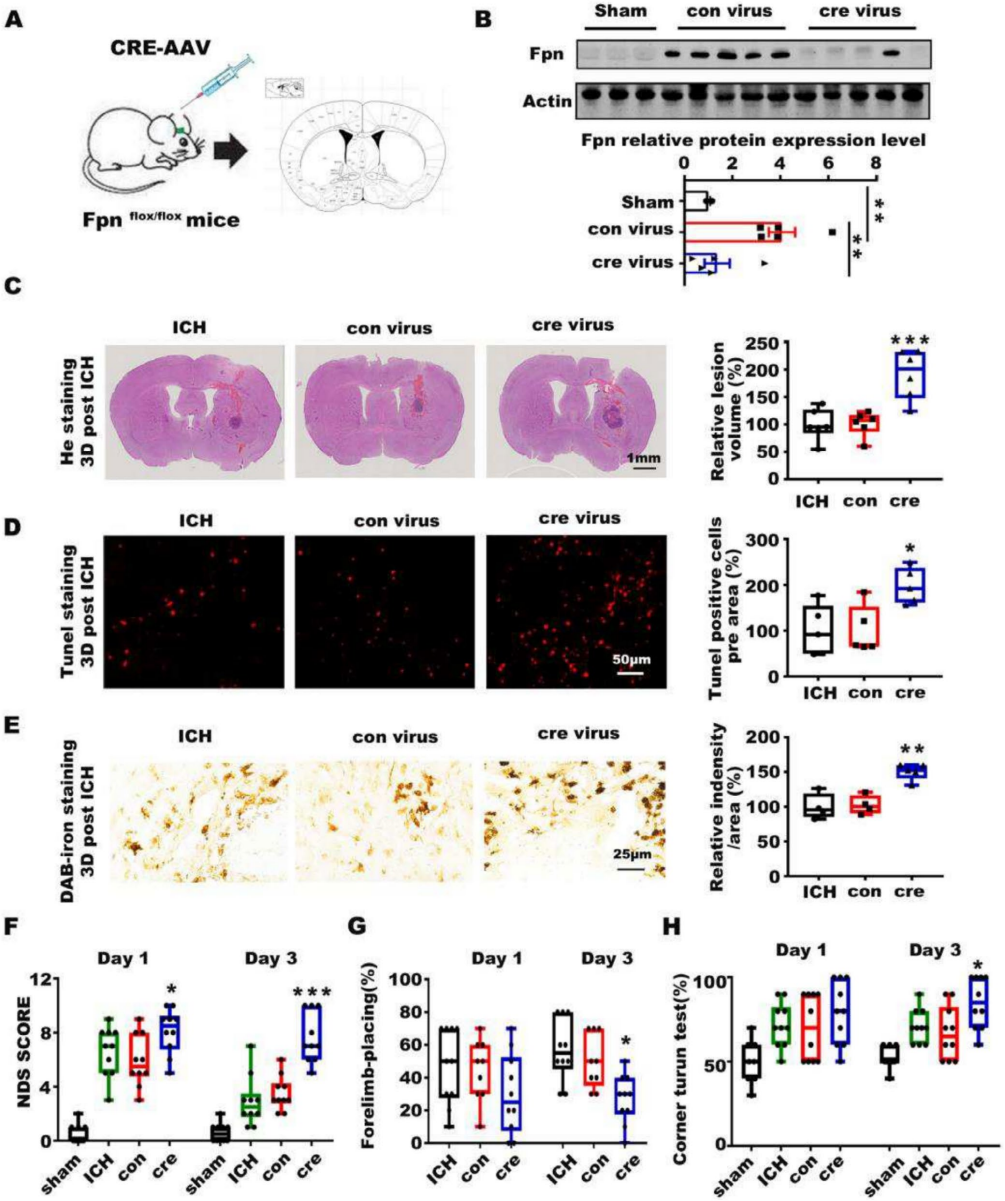

The authors apologize for this error.

