# Supplementary material for: Targeting miR‐124/Ferroportin signaling ameliorated neuronal cell death through inhibiting apoptosis and ferroptosis in aged intracerebral hemorrhage murine model
Source: Aging Cell. 2020 Oct 17;19(11):e13235. doi: 10.1111/acel.13235 (PMC7681046; doi:10.1111/acel.13235)
Supplement: Supplementary file 1 [file ACEL-19-e13235-s001.docx]

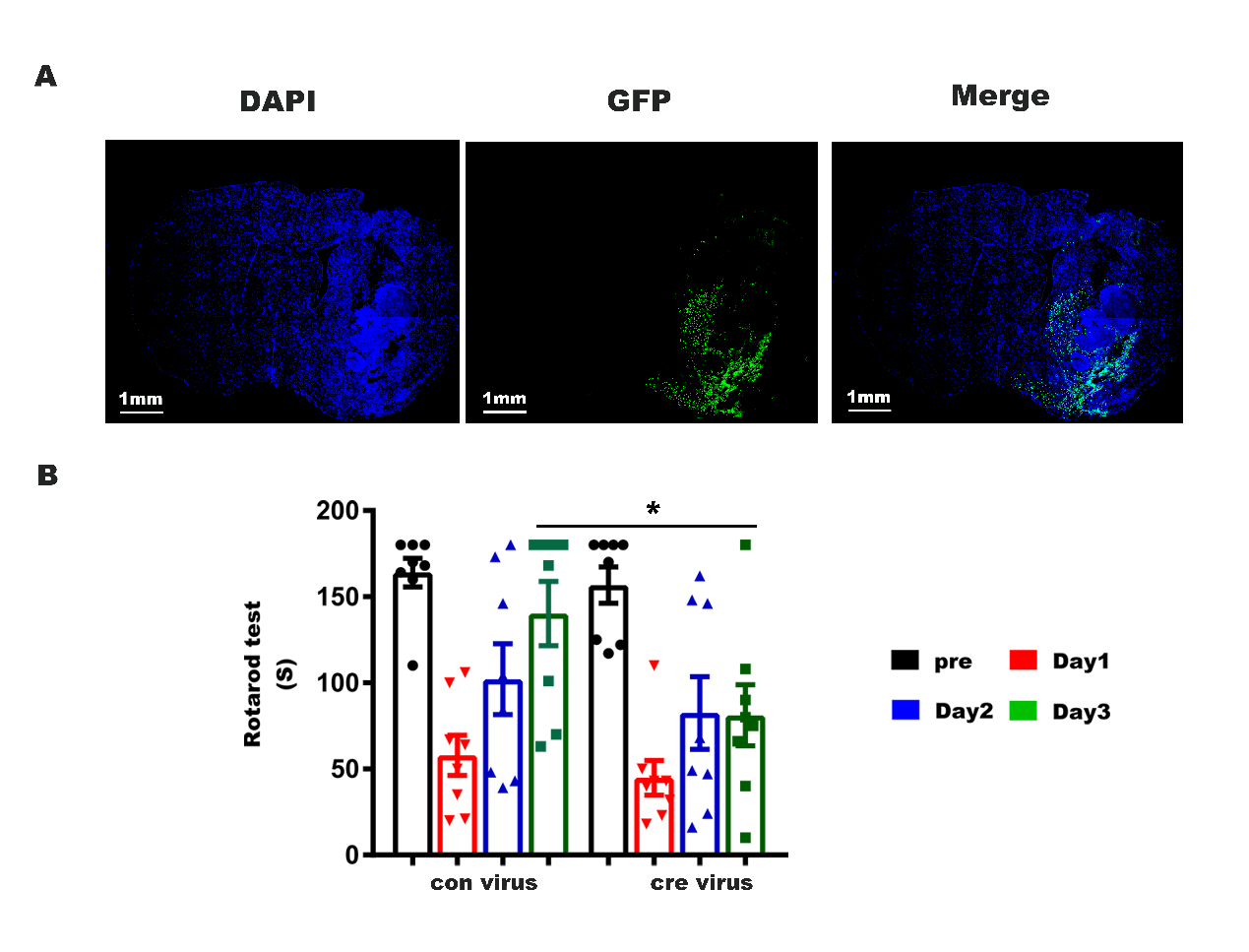


**Supplementary Figure 1. Deficient of Fpn decreased the time spent on rotarod test of the mice post-ICH.**

(A) Representative immuno-fluorescence staining of brain slice from the mice post-ICH which were pre-injected with AAV virus. Sections were stained for DAPI and the AAV were labeled with GFP. (B) The time spent on the rotarod test of the mice after ICH which were pre-injected with cre virus or con virus (n=8).

Data are shown as the mean ± SEM. Statistical analyses were carried out using two-way ANOVA. *p < 0.05

**
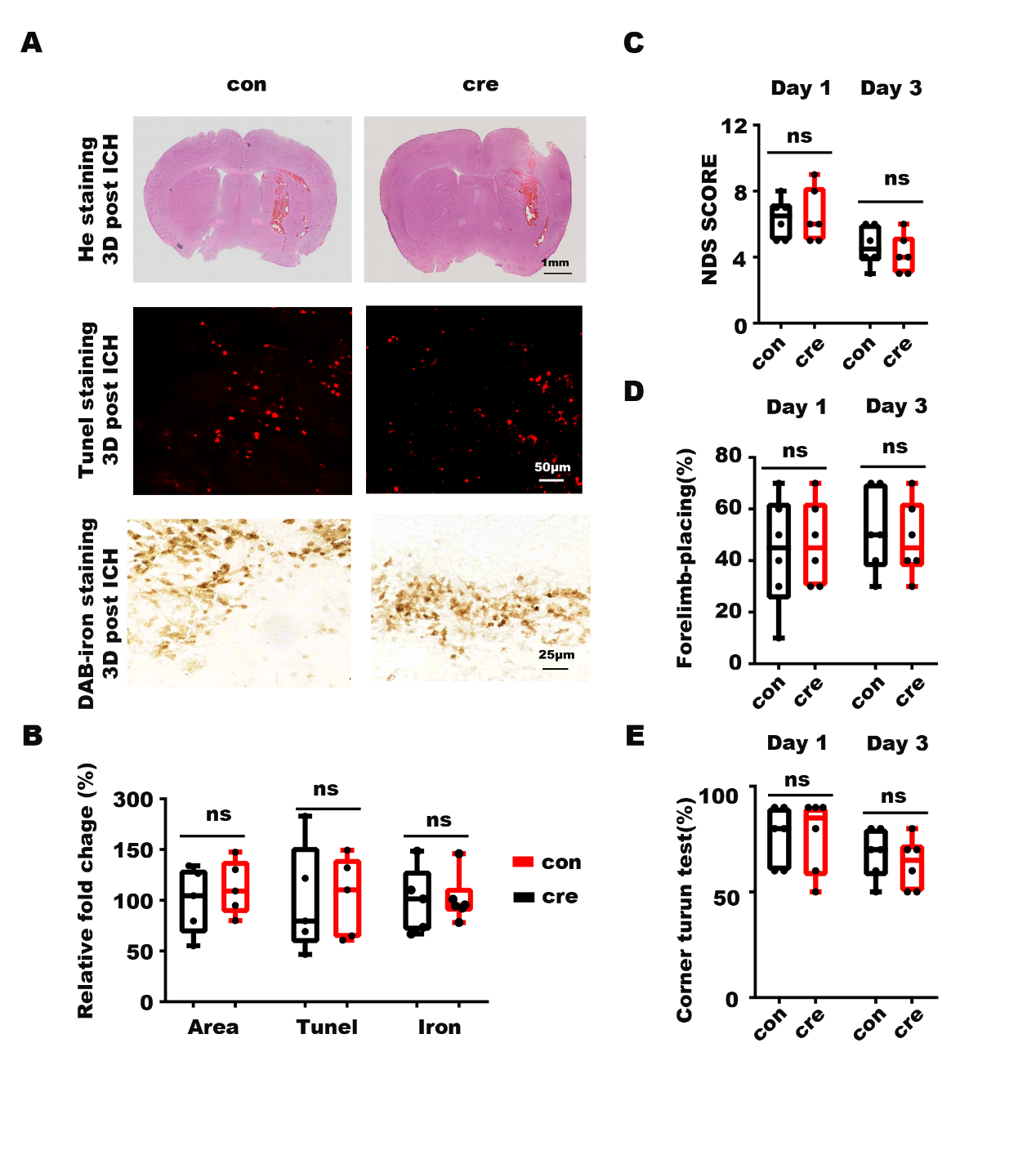
**

**Supplementary Figure 2. AAV-cre injection had no effect on the outcome of ICH in non-floxed C57 mice**

(A) Brain sections from these two groups were subjected to HE staining (hematoxylin and eosin), TUNEL staining and Perls’ blue iron staining (DAB-enhanced). Con, non-floxed C57 mice injected with control AAV; Cre, non-floxed C57 mice injected with Cre AAV (n=5, per group). (B) The quantification of the pictures above, and the results are shown as the fold change (%) of the control. (C) Neurologic deficit score, (D) Forelimb-placing capacity and (E) corner turn test for all these mice post-ICH (n=6, per group).

The results are shown as box-and-whisker plots (the middle horizontal line within the box represents the median, the boxes extend from the 25th to the 75th percentile, and the whiskers represent 95% confidence intervals).

Statistical analyses were carried out using multiple t-test and two-way ANOVA .


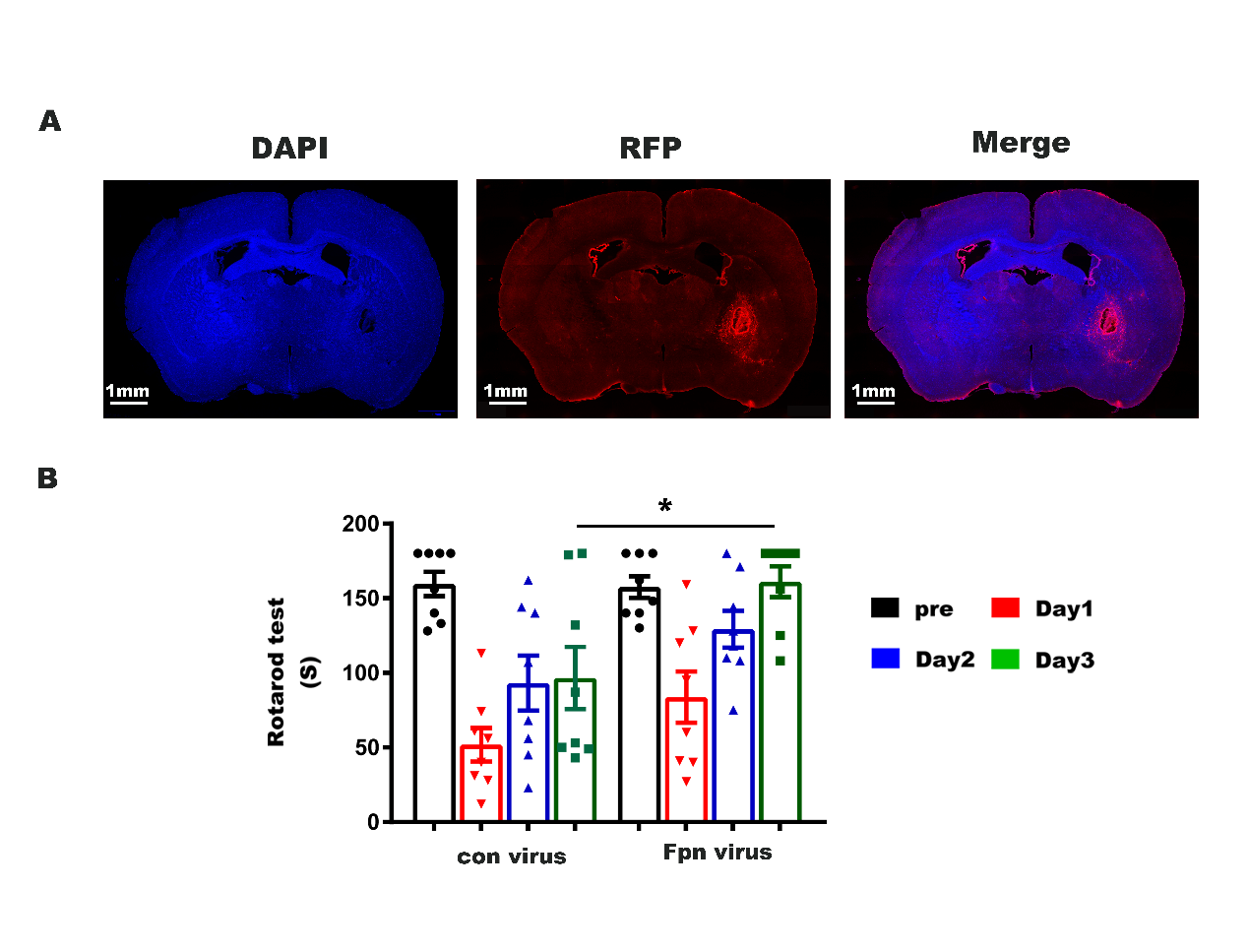


**Supplementary Figure 3. Overexpression of Fpn ameliorate the time spent on rotarod test of the mice after ICH.**

(A) Representative immuno-fluorescence staining of brain slice from the mice post-ICH which were pre-injected with Fpn-expressed AAV virus (Fpn virus) or control AAV virus (con virus). Sections were stained for DAPI and the AAV were labeled with m-cherry. (B) The time spent on the rotarod test of these mice (n=8).

Data are shown as the mean ± SEM of at least 3 independent experiments. Statistical analyses were carried out using two-way ANOVA. *p < 0.05


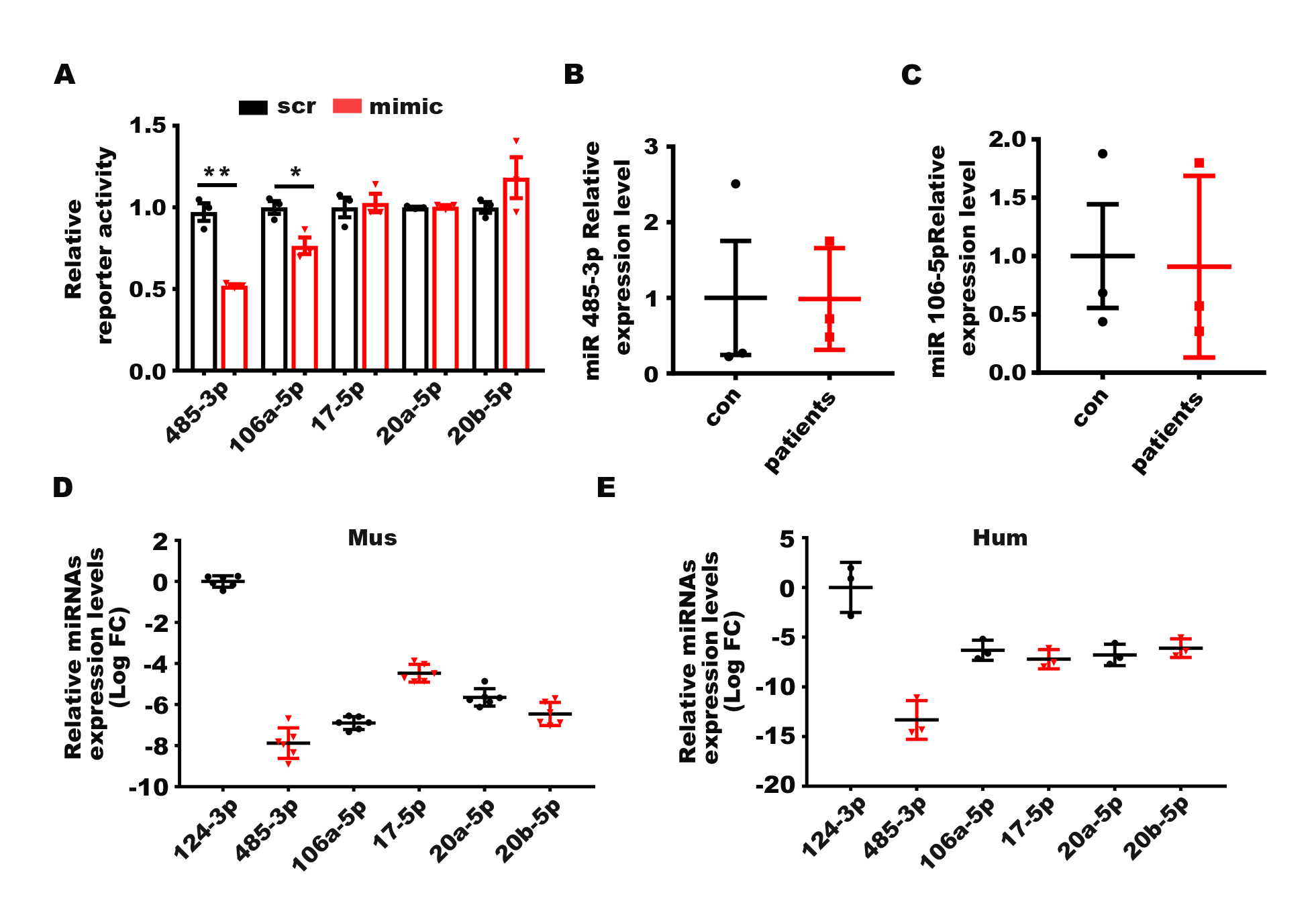


**Supplementary Figure 4. miR-485-3p and miR-106a-5p could inhibit the activity of 3’UTR of Fpn in vitro**

(A) 293T cells were cotransfected with mimic of miRs (miR-485-3p, miR -106a-5p, miR-17-5p, miR-20a-5p, miR-20b-5p) and reporter plasmids obtained 3’UTR of murine Fpn. Relative luciferase activity was assessed, paired scrambles were cotransfected as controls, and results are shown as fold change of the controls. (B) Expression levels of miR 485-3p in the tissues of ICH patients (n=3) and controls (n=3). (C) Expression levels of miR 106a-5p in the tissues of ICH patients (n=3) and controls (n=3). (D) Expression levels of miRs (miR 485-3p, miR 106a-5p, miR 17-5p, miR 20a-5p, miR 20b-5p) compared to miR-124-3p in murine brain tissues. (E) Expression levels of miRs (miR 485-3p, miR 106a-5p, miR 17-5p, miR 20a-5p, miR 20b-5p) compared to miR-124-3p in human brain tissues.

Data are shown as the mean ± SEM of at least 3 independent experiments. Statistical analyses were carried out using mutiple t-test. *p < 0.05; **p < 0.01;

**
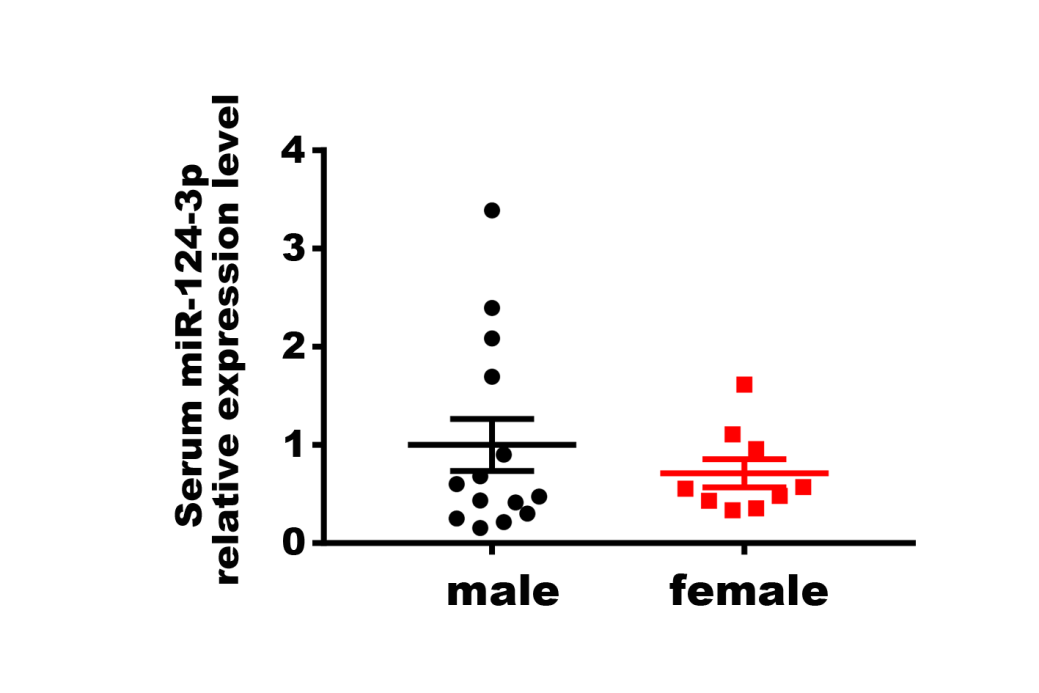
**

**Supplementary Figure 5. Serum miR-124 level was not correlated with gender.**

Correlation analysis between the serum miR-124 level and the gender of all the patients or healthy controls. (male, n=14; female. n=9)

**
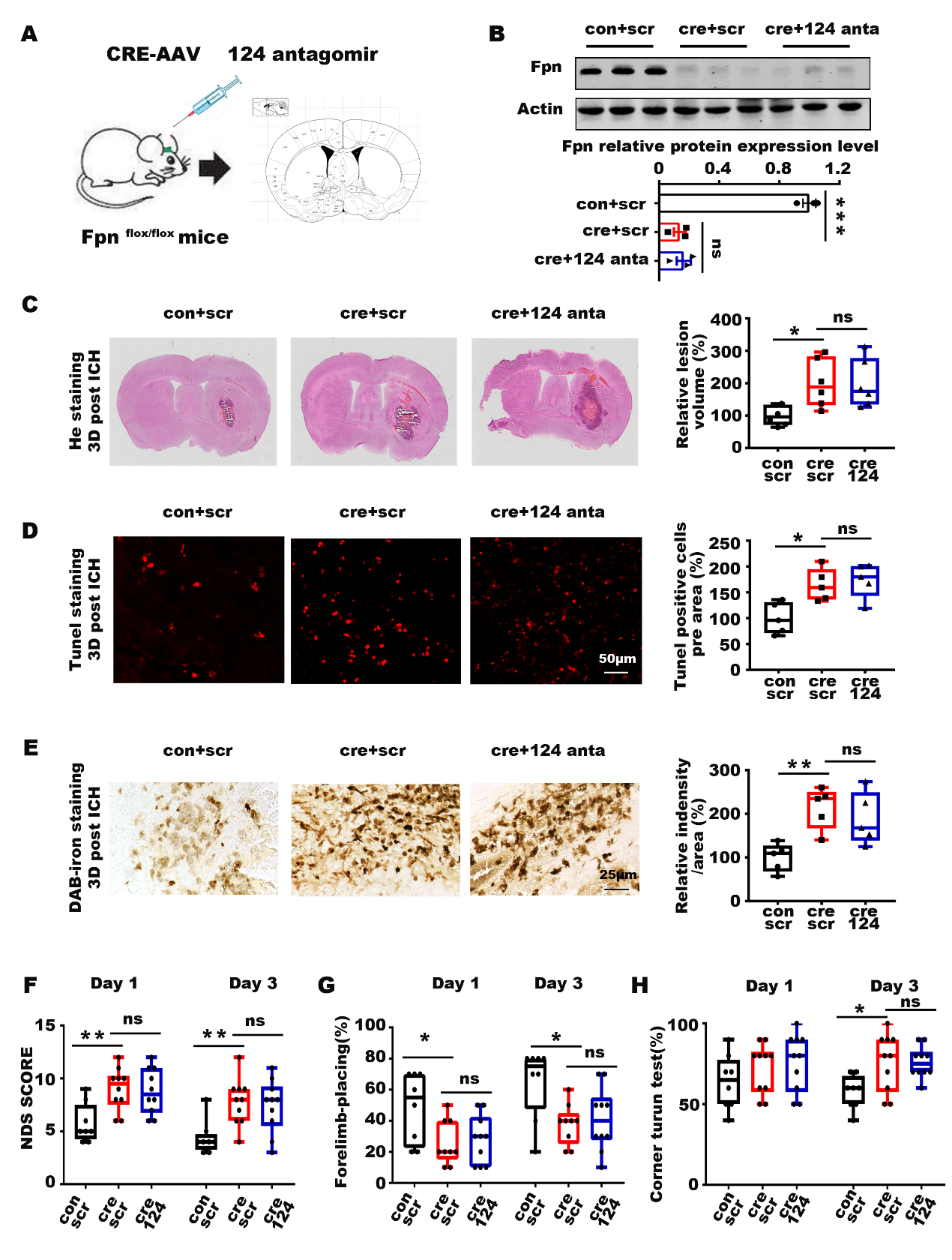
**

**Supplementary Figure 6. Fpn deficiency abolished the alleviation of the symptoms of ICH induced by antagomir of miR-124.**

(A) Cartoon outline of the stereotactic injection procedure in aged Fpn floxed mice. (B) Protein level of Fpn in the brain tissues of the ICH model mice injected with the cre-expression AAV and antagomir of miR-124 (cre 124), cre-expression AAV and scrambles (cre scr), control AAV and scrambles (con scr). (C) Brain sections were stained with hematoxylin and eosin (left), and the lesion volume was calculated (right). The results are shown as the fold change (%) of the controls. Con scr, mice injected with control AAV and scrambles before the ICH model was generated. Cre scr, mice injected with cre-expression AAV and scrambles before the ICH model was generated. Cre 124, mice injected with cre-expression AAV and antagomir of miR-124 before the ICH model was generated (n=6). (D) Sections were subjected to TUNEL staining (left), quantification is shown (right), and the results are shown as the fold change (%) of the control (n=5). (E) Perls’ staining (left) and quantification of the staining intensity of cells (right) and the results are shown as the fold change (%) of the control (n=5). (F) Neurologic deficit score, (G) Forelimb-placing capacity and (H) corner turn test for all these mice post-ICH (n=8-10, per group).

The results are shown as box-and-whisker plots (the middle horizontal line within the box represents the median, the boxes extend from the 25th to the 75th percentile, and the whiskers represent 95% confidence intervals).

Statistical analyses were carried out using one-way and two-way ANOVA . *p < 0.05; **p < 0.01.

**Supplementary Experimental Procedures**

**Western Blot Analysis**

Proteins were separated as described previously ([Wang et al., 2018](#_ENREF_5)). Briefly, tissues were homogenized with sample buffer (pH 7.6, 50 mM Tris-HCl, 10 mM dithiothreitol, 2% sodium dodecyl sulfate, 10% glycerol, and 0.2% bromophenol blue) on ice and boiled for 10 minutes. Then, the proteins were separated by 10% SDS-PAGE gel and transferred to nitrocellulose membranes. The membranes were incubated with primary antibodies overnight at 4°C, followed by incubation with anti-rabbit or anti-mouse IgG conjugated secondary antibodies (LI-COR, Lincoln, NE, USA) for 1 h at room temperature and detected using an Odyssey Imaging System (LI-COR, Lincoln, NE, USA). The proteins were probed with antibodies against Slc40A1 (Fpn1, Novus Biologicals), Cox2, actin, Erk1/2 (Proteintech), p-Erk1/2 (CST), p-MLKL (Abcam), and cleaved caspase3 (CST). Detailed information about the antibodies is available in **Supplementary Table 1**.

**Quantitative RT-PCR**

Total RNA was extracted using TRIzol reagent (Invitrogen), and 1 μg RNA was reverse transcribed ([Wang et al., 2018](#_ENREF_5)). miRNA was extracted from tissues and serum using a miRcute miRNA Isolation Kit (TIANGEN Biotech, Beijing, catalog DP501) according to the manufacturer’s instructions. miRNAs and other noncoding RNAs were polyadenylated by poly(A) polymerase and subsequently converted into cDNA by reverse transcriptase with oligodT priming using a miRcute miRNA First-Strand cDNA kit (TIANGEN Biotech, Beijing, catalog KR201). qRT-PCR was performed on an ABI StepOne Plus using SYBR Green ® Premix Ex Taq (Takara, Tokyo, Japan). The qPCR data were shown as relative mRNA expression versus control group. The results showed the fold-change (2^-delta /delta Ct^) of relative expression versus control group (-delta /delta Ct = -{sample (CT gene-CT actin)- con(CT gene-CT actin)}). For mRNA, the gene expression was normalization to β-actin. For miRNA, the gene expression was normalization to U6. The primer sequences are detailed in **Supplementary Table 2**.

**Immunofluorescence and Immunohistochemistry**

Mice were anesthetized and immediately perfused continuously with normal saline and 4% paraformaldehyde solution. The brains were dissected and postfixed for 24 h at 4°C, and 25-μm slices of brain tissues were cut with a microtome. The procedure was described previously ([Liu et al., 2017](#_ENREF_3)). TUNEL staining was performed using an In Situ Cell Death Detection kit (Roche, catalog 12156792910, 11684817910) according to the manufacturer’s instructions. The slices were imaged using a laser confocal microscope (LSM780, Carl Zeiss). For Immunohistochemistry staining, DAB- enhanced Perls’ staining was used to detect iron accumulation as previously described ([Donovan et al., 2005](#_ENREF_2)). Sections of brain tissue were washed with PBS and H2O2 and incubated with freshly prepared Perls’ solution (5% potassium ferrocyanide [Sigma-Aldrich]/10% hydrochloric acid) for 1 h, followed by PBS washes for 3 times. Then, DAB incubation was carried out for 3 minutes. Hematoxylin and eosin (HE) staining was performed according to the manufacturer’s instructions (Wuhan Goodbio Technology, catalog G1005-1, G1005-2). The images were observed under a microscope (Olympus BX60, Tokyo, Japan).

**Virus and Stereotaxic Injection**

Mice were treated with a mixture of ketamine (100 mg/kg) and dexmedetomidine (0.5 mg/kg). Holes were drilled above the caudate nucleus (bregma 0: 0.8 mm anterior, 2 mm left lateral and 3.5 mm deep). Antagomirs, scrambles (100μM, 2 μL), or AAVs (10^12^ IU/μL, 2 μL) were microinfused into the caudate nucleus via a cannula connected to a Hamilton (Reno, NV) microsyringe. The infusion rate was 0.2 μL/min, and the cannula was left in place for 10 minutes following completion of the infusion. Antagomir of miR-124 and relative scrambles were injected into C57 two weeks before the ICH model. AAVs were injected into the mice one month before ICH model. miR-124 inhibitors/antagomirs and the scrambled control were purchased from RiboBio (Guangzhou, China). Adeno-associated viruses (AAVs) for Fpn and cre overexpression, were purchased from NeuronBiotech (Shanghai,China).

**Luciferase Activity Assay**

Cells were harvested, and lysates were used to detect firefly and Renilla luciferase activities using a dual luciferase reporter assay kit (Promega) according to the manufacturer’s protocol. The normalized values (Renilla/firefly activity) were used for analysis. The experiments were performed in triplicate. miR-124 overexpression construct was obtained from GeneCopoeia (Rockville, MD). 3’-UTR of mouse Fpn was amplified with the following primers: Forward 5’-CCCTCGAGAAAT AGTTTAGCTGTGGCCCCTG-3’, reverse:5’-TTGCGGCCGCTAAGTAACAATAAT TGGGAGAACAC-3’. 3’-UTR of human Fpn was amplified with the following primers: forward 5’ -CCCTCGAGTTTAACTGTTGCTATCCTGTTACT-3’, reverse 5’-TTGCGGCCGCC CTTTTTACAAAGATTTTACAACATAG-3’. All the mimics and scrambled control were purchased from RiboBio (Guangzhou, China). The polymerase chain reaction fragment was inserted into psiCHECK2 (Promega, Madison, WI). Mutation was performed using a fast mutation kit from Agilent (Brighton, MA).

**Neurological Deficit Scores**

Following blood injection, the animals were scored neurologically for focal deficits at 24 and 72 h with the use of a 28-point neurological scoring system that has been shown to be sensitive to focal motor and sensory deficits after injury ([Clark, Lessov, Dixon, & Eckenstein, 1997](#_ENREF_1); [Rynkowski et al., 2008](#_ENREF_4)).

**Forelimb placing test**

Briefly, for the forelimb placing test, the animals were gently held by their torsos, and each forelimb was independently tested by brushing the respective vibrissae on the corner edge of a countertop. Each mouse was tested ten times for each forelimb, and the percentage of trials in which the mouse placed the appropriate forelimb on the edge of the countertop in response to the vibrissae stimulation was recorded. All data were recorded by an observer blinded to the experimental cohort.

**Corner Turn Test**

For the corner turn test, the mouse was allowed to walk down a hall into a 30° corner. The mouse’s choice of turn direction was noted. The number of right and left turns out of ten total attempts was recorded. All data were recorded by an observer blinded to the experimental cohort.

**Rotarod Test**

For the rotarod test, the mice were trained for 3 days before the autologous blood infusion, and the mice were habituated to stay on the stationary drum. Habituation was repeated 3 times every day, and the rotation was set at a relatively slow speed to make the task easier for the learning animals. The latency on the third day was used as a basic control. Then, the latency to falling was recorded at 24, 48 and 72 h after hemorrhage, and the latency on the rod on each day were analyzed.

**Reference**

Clark, W. M., Lessov, N. S., Dixon, M. P., & Eckenstein, F. (1997). Monofilament intraluminal middle cerebral artery occlusion in the mouse. *Neurological Research, 19*(6), 641-648.

Donovan, A., Lima, C. A., Pinkus, J. L., Pinkus, G. S., Zon, L. I., Robine, S., & Andrews, N. C. (2005). The iron exporter ferroportin/Slc40a1 is essential for iron homeostasis. *Cell Metab, 1*(3), 191-200. doi:10.1016/j.cmet.2005.01.003

Liu, D., Tang, H., Li, X. Y., Deng, M. F., Wei, N., Wang, X., . . . Zhu, L. Q. (2017). Targeting the HDAC2/HNF-4A/miR-101b/AMPK Pathway Rescues Tauopathy and Dendritic Abnormalities in Alzheimer's Disease. *Mol Ther, 25*(3), 752-764. doi:10.1016/j.ymthe.2017.01.018

Rynkowski, M. A., Kim, G. H., Komotar, R. J., Otten, M. L., Ducruet, A. F., Zacharia, B. E., . . . Connolly, E. S. (2008). A mouse model of intracerebral hemorrhage using autologous blood infusion. *Nature Protocols, 3*(1), 122-128. doi:10.1038/nprot.2007.513

Wang, X., Liu, D., Huang, H. Z., Wang, Z. H., Hou, T. Y., Yang, X., . . . Zhu, L. Q. (2018). A Novel MicroRNA-124/PTPN1 Signal Pathway Mediates Synaptic and Memory Deficits in Alzheimer's Disease. *Biological Psychiatry, 83*(5), 395-405. doi:10.1016/j.biopsych.2017.07.023

**Supplementary TABLE 1. Antibody information related to experimental procedures, with catalog number/manufacturer, and used dilutions**

| **Antibody** | **Catalog number/manufacturer** | **Dilution** | **Reference** |
| --- | --- | --- | --- |
| Fpn | NBP1-21502, Novus Biologicals | 1:1000 for wb | PMID:24658075, 31585094，23102618，29599243 |
| P-Erk1/2 | 4370，Cell Signaling Technology | 1:1000 for wb | PMID:30568162 |
| Erk 1/2 | 16443-1-AP, Proteintech | 1:1000 for wb | PMID:27267806 |
| Cox2 | 12375-1-AP, Proteintech | 1:1000 for wb | PMID:31636012 |
| p-mlkl | 196436, Abcam | 1:1000 for wb | PMID:29920190 |
| Cleaved-caspase3 | 9661, Cell Signaling Technology | 1:1000 for wb | PMID: 31537808 |
| Beta Actin | 66009-1-Ig ,Proteintech | 1:3000 for wb | PMID:24030155 |

**Supplementary TABLE 2. Sequences of oligonucleotide primers**

| **Target gene** | **Species** | **primer sequence** |
| --- | --- | --- |
| Actin forward | hum | TGGCACCCAGCACAATGAA |
| Actin reverse | hum | CTAAGTCATAGTCCGCCTAGAAGCA |
| Fpn forward | hum | CTACTTGGGGAGATCGGATGT |
| Fpn reverse | hum | CTGGGCCACTTTAAGTCTAGC |
| Actin forward | mus | GAGACCTTCAACACCCCAGC |
| Actin reverse | mus | GGAGAGCATAGCCCTCGTAGAT |
| Fpn forward | mus | GTCGGCCAGATTATGACATTTG |
| Fpn reverse | mus | ATTCCAACCGGAAATAAAACC |
| ATPG53 forward | mus | TCTGCATCAGTGTTATCTCGGC |
| ATPG53 reverse | mus | CACCAGAACCAGCAACTCCTA |
| IREB2 forward | mus | TTCTGCCTTACTCAATACGGGT |
| IREB2 reverse | mus | AGGGCACTTCAACATTGCTCT |
| CS forward | mus | GGACAATTTTCCAACCAATCTGC |
| CS reverse | mus | TCGGTTCATTCCCTCTGCATA |
| RPL8 forward | mus | AAGGCGCGGGTTCTGTTTT |
| RPL8 reverse | mus | GCTCTGTCCGCTTCTTGAATC |
| PTGS2 forward | mus | TTCAACACACTCTATCACTGGC |
| PTGS2 reverse | mus | AGAAGCGTTTGCGGTACTCAT |

**Supplementary TABLE 3. Detailed statistical analysis information**

| **Figure and nr of animals or cells used** | **Statistical analysis** | **Post hoc Tukey’s test**  **or**  **mutiple t-test** | **Mean ± SEM** |
| --- | --- | --- | --- |
| **Figure 1B**  CON (n=4)  IPS (n=4) | t-test | IPS versus CON  P=0.0041 | Fold change  (IPS versus CON)  4.763 ± 0.7907 |
| **Figure 1C**  CON (n=6)  IPS (n=6) | t-test | IPS versus CON  P=0.0002 | Fold change  (IPS versus CON)  2.176 ± 0.1968 |
| **Figure 1E**  CON (n=4)  Patient (n=4) | t-test | Patient versus CON  P=0.0274 | Fold change  (Patient versus CON)  2.011 ± 0.1339 |
| **Figure 1F**  CON (n=4)  Patient (n=4) | t-test | Patient versus CON  P=0.2668 | Fold change  (Patient versus CON)  20.63 ± 16.04 |
| **Figure 2B**  Sham (n=3)  con (n=5)  cre (n=5) | One-way ANOVA  F (2, 10) = 10.85 | Sham vs Con, P=0.0075  Cre vs Con, P=0.0067 | Sham, 1 ± 0.07196  Con, 4.068 ± 0.549  Cre, 1.365 ± 0.524 |
| **Figure 2C**  ICH (n=6)  con (n=6)  cre (n=6) | One-way ANOVA  F (2, 15) = 14.79 | con vs ICH, P=0.9968  cre vs con, P=0.0008 | Fold change (%)  ICH, 100 ± 11.71  Con, 101.5 ± 9.15  Cre, 191.2 ± 18.23 |
| **Figure 2D**  ICH (n=5)  con (n=5)  cre (n=5) | One-way ANOVA  F (2, 12) = 6.519 | con vs ICH, P=0.9994  cre vs con, P=0.0227 | Fold change (%)  ICH, 100 ± 24.6  Con, 101 ±23.37  cre, 198 ± 17.49 |
| **Figure 2E**  ICH (n=4)  con (n=5)  cre (n=5) | One-way ANOVA  F (2, 11) = 13.57 | con vs ICH, P=0.7282  cre vs con, P=0.0040 | Fold change (%)  ICH, 100 ± 9.396  Con, 108.3 ± 7.803  Cre, 150.7 ± 5.273 |
| **Figure 2F**  Sham (n=10)  ICH (n=10)  con (n=10)  cre (n=10) | Two-way ANOVA  Row Factor  F (3, 72) = 74.74, P<0.001  Column Factor  F (1, 72) = 21.99, P<0.001 | Day1  Sham vs ICH, P<0.0001  Con vs ICH, P=0.7441  Cre vs Con, P=0.0116  Day3  Sham vs ICH, P=0.0076  Con vs ICH, P=0.8224  Cre vs Con, P<0.0001 | Day1  Sham 0.5±0.224  Con 6.6 ±0.581  ICH 5.9±0.605  Cre 8.1 ±0.526  Day3  Sham 0.6±0.221  Con 2.9±0.586  ICH 3.5±0.401  Cre 7.6±0.581 |
| **Figure 2G**  ICH (n=10)  con (n=9)  cre (n=10) | Two-way ANOVA  Row Factor  F (2, 52) = 7.564, P=0.0013  Column Factor  F (1, 52) = 0.6442, P=0.4259 | Day1  Con vs ICH, P=0.9894  Cre vs Con, P=0.3162  Day3  Con vs ICH, P=0.8298  Cre vs Con, P=0.0434 | Day1  ICH 47±7.31  Con 44.44±6.26  Cre 30±7.75  Day3  ICH 57±5.97  Con 50±55.3  Cre 27±4.73 |
| **Figure 2H**  Sham (n=10)  ICH (n=10)  con (n=10)  cre (n=10) | Two-way ANOVA  Row Factor  F (3, 72) = 16.17, P<0.001  Column Factor  F (1, 72) = 0.09756, P=0.7557 | Day1  Sham vs ICH, P=0.0053  Con vs ICH, P=0.9894  Cre vs Con, P=0.5001  Day3  Sham vs ICH, P=0.0208  Con vs ICH, P=0.9238  Cre vs Con, P=0.0316 | Day1  Sham 50±3.651  Con 72±4.163  ICH 70±6.146  Cre 79±5.859  Day3  Sham 52±2  Con 71±3.145  ICH 67±4.955  Cre 85±4.773 |
| **Figure 3B**  Sham (n=3)  con (n=5)  Fpn (n=5) | One-way ANOVA  F (2, 10) = 15.62 | Sham vs Con, P=0.0286  Fpn vs Con, P=0.0406 | Fold change  Con, 2.497 ± 0.2948  Fpn,3.703 ± 0.3553 |
| **Figure 3C**  ICH (n=6)  con (n=6)  Fpn (n=6) | One-way ANOVA  F (2, 15) = 6.212 | con vs ICH, P=0.9988  Fpn vs con, P=0.0198 | Fold change (%)  ICH, 100 ± 6.56  Con, 102.4 ± 5.613  Fpn, 64.34 ± 12.46 |
| **Figure 3D**  ICH (n=5)  con (n=5)  fpn (n=5) | One-way ANOVA  F (2, 12) = 11.15 | con vs ICH, P=0.8752  Fpn vs con, P=0.0064 | Fold change (%)  ICH, 100 ± 9.973  Con, 93.35 ± 12.33  Fpn, 42.02 ± 4.402 |
| **Figure 3E**  ICH (n=5)  con (n=5)  fpn (n=5) | One-way ANOVA  F (2, 12) = 9.96 | con vs ICH, P=0.4806  Fpn vs con, P=0.0220 | Fold change (%)  ICH, 100 ± 10.74  Con, 85.58 ± 6.465  Fpn, 47.69 ± 7.926 |
| **Figure 3F**  Sham (n=10)  ICH (n=10)  con (n=10)  Fpn (n=10) | Two-way ANOVA  Row Factor  F (3, 72) = 64.8, P<0.001  Column Factor  F (1, 72) = 28.6, P<0.001 | Day1  Sham vs ICH, P<0.0001  Con vs ICH, P=0.6361  Fpn vs Con, P=0.9566  Day3  Sham vs ICH, P<0.0001  Con vs ICH, P=0.2511  Fpn vs Con, P=0.0163 | Day1  Sham 0.7±0.213  Con 6.1±0.605  ICH 6.8±0.467  Fpn 6.5±0.373  Day3  Sham 0.8±0.249  Con 4.2±0.554  ICH 5.3±0.423  Fpn 3.5±0.269 |
| **Figure 3G**  ICH (n=10)  con (n=10)  fpn (n=10) | Two-way ANOVA  Row Factor  F (2, 54) = 0.5576, P=0.5758  Column Factor  F (1, 54) = 0.5718, P=0.4528 | Day1  Con vs ICH, P=0.5772  Fpn vs Con, P=0.8511  Day3  Con vs ICH, P=0.9885  Cre vs Con, P=0.8511 | Day1  Con 41±5.47  ICH 49±4.58  Cre 44±6  Day3  Con 50±3.94  ICH 49±5.04  Cre 44±3.71 |
| **Figure 3H**  Sham (n=10)  ICH (n=10)  con (n=10)  Fpn (n=10) | Two-way ANOVA  Row Factor  F (3, 72) = 13.33, P<0.001  Column Factor  F (1, 72) =1.722, P=0.1936 | Day1  Sham vs ICH, P=0.0011  Con vs ICH, P=0.9877  Fpn vs Con, P=0.2096  Day3  Sham vs ICH, P=0.0137  Con vs ICH, P=0.8448  Fpn vs Con, P=0.0137 | Day1  Sham 52±2.906  Con 76±4.522  ICH 74±4.761  Cre 62±5.538  Day3  Sham 50±3.651  Con 69±4.819  ICH 74±4.522  Cre 55±3.073 |
| **Figure4B** |  |  | Fold change  (Ips versus con) |
| con (n=5) | Mutiple t-test |  |  |
| Ips (n=5) |  | 93-5p, con vs Ips , P=0.8099 | 93-5p, 1.04±01325 |
|  |  | 485-3p, con vs Ips , P=0.0126 | 485-3p, 0.565±0.057 |
|  |  | 124, con vs Ips , P=0.0024 | 124, 0.559±0.044 |
|  |  | 106b-5p, con vs Ips, P=0.7401 | 106b-5p, 0.947±0.121 |
|  |  | 106a-5p, con vs Ips ,P=0.0033 | 106a-5p, 1.769±0.180 |
|  |  | 17-5p, con vs Ips , P=0.0055 | 17-5p, 1.524±0.123 |
|  |  | 20a-5p, con vs Ips, P=0.0091 | 20a-5p, 1.910±0.258 |
|  |  | 20b-5p, con vs Ips , P=0.0054 | 20b-5p, 1.709±0.185 |
| **Figure4C**  CON (n=4)  Patient (n=4) | t-test | Patient versus CON  P=0.0431 | Fold change  (Patient versus CON)  Con: 1 ± 0.3882  Patient : 0.0098 ± 0.0089 |
| **Figure 4E**  **Mus 3’UTR**  **124 vs Scr** | Two-way ANOVA | MUS 124 vs Scr | Fold change  (124 vs Scr) |
| WT (n=3vs3)  Mutant-1(n=3vs3)  Mutant-2 (n=3vs3)  **Hum 3’UTR**  **124 vs Scr**  WT (n=3vs3)  Mutant-1(n=3vs3) | Row Factor  F (2, 12) = 88.1, P<0.0001  Column Factor  F (1, 12) =58.76, P<0.0001  Row Factor  F (1, 8) = 54.12, P<0.0001  Column Factor  F (1, 8) =14.44, P=0.0052 | WT, P<0.0001  Mutant-1, P=0.9952  Mutant-2, P=0.6864  HUM 124 vs Scr  WT, P=0.0003  Mutant, P=0.2978 | WT, 0.5048±0.0169  Mutant-1, 1.016±0.0213  Mutant-1, 1.048±0.0270  HUM 124 vs Scr  WT, 0.6454±0.0108  Mutant, 1.113±0.0410 |
| **Figure 4F**  Scr (n=3)  124 anta (n=3) | t-test | P=0.0428 | Scr, 1 ± 0.1164  124 anta, 1.834 ± 0.26 |
| **Figure 4G**  Scr (n=3)  124 mimic (n=3) | t-test | P=0.0367 | Scr, 1 ± 0.06995  124 mimic, 0.7799 ± 0.01379 |
| **Figure 4I**  CON (n=16)  Patient (n=8) | t-test | Patient versus CON  P=0.0445 | Fold change  (Patient versus CON)  Patient : 0.3733 ± 0.0611 |
| **Figure 4J** |  |  |  |
| Human sample(n=8) | Linear regression | r^2^=0.6609 |  |
|  | Y = 2.887*X +4.376 | P=0.0472 |  |
| **Figure 4K** |  |  |  |
| Human sample(n=8) | Linear regression | r^2^=0.5081 |  |
|  | Y = 0.858*X +0.528 | P=0.0141 |  |
| **Figure 4L**  Human sample(n=8) | Linear regression  Y =3.137*X +16.72 | r^2^=0.3691  P=0.1101 |  |
| **Figure 5B**  ICH (n=5)  Con (n=6)  124 (n=6) | Multiple t-test | Scr vs ICH, P=0.9528  124 vs scr, P=0.0002 | Fold change  ICH, 0.5587 ± 0.044  scr, 0.6842± 0.014  124, 0.1025 ± 0.036 |
| **Figure 5C**  ICH (n=6)  scr (n=6)  124 (n=6) | One-way ANOVA  F (2, 15) = 5.982 | scr vs ICH, P=0.8651  124 vs scr, P=0.0408 | Fold change (%)  ICH, 100 ± 9.322  scr, 93.7 ± 9.25  124, 60.61 ± 7.21 |
| **Figure 5D**  ICH (n=5)  Scr (n=5)  124 (n=5) | One-way ANOVA  F (2, 12) = 6.458 | scr vs ICH, P=0.7812  124 vs scr, P=0.0136 | Fold change (%)  ICH, 100 ± 11.45  Scr, 108.8 ± 8.547  124, 64.79 ± 6.878 |
| **Figure 5E**  ICH (n=5)  Scr (n=5)  124 (n=5) | One-way ANOVA  F (2, 12) = 3.722 | Scr vs ICH, P=0.7899  124 vs con, P=0.1642 | Fold change (%)  ICH, 100 ± 14.19  Scr, 90.64 ± 6.847  124, 62.83 ± 7.271 |
| **Figure 5F**  Sham (n=10)  ICH (n=10)  Scr (n=10)  124 (n=10) | Two-way ANOVA  Row Factor  F (3, 72) =35.76, P<0.001  Column Factor  F (1, 72) = 21.56, P<0.001 | Day1  Sham vs ICH, P<0.0001  Scr vs ICH, P=0.9999  124 vs Scr, P=0.8178  Day3  Sham vs ICH, P=0.0013  Scr vs ICH, P=0.3713  124 vs Scr, P=0.0134 | Day1  Sham 0.8 ±0.2  Scr 7.1±0.737  Scr 7.1±0.674  124 6.4±0.792  Day3  Sham 0.9±0.18  Scr 4±0.745  Scr 5.3±0.496  124 2.8±0.249 |
| **Figure 5G**  ICH (n=10)  Scr (n=10)  cre (n=10) | Two-way ANOVA  Row Factor  F (2, 54) =2.664, P=0.0802  Column Factor  F (1,54) = 4.281, P=0.0433 | Day1  Scr vs ICH, P=0.8371  124 vs Scr, P=0.1230  Day3  Con vs ICH, P=0.4731  124 vs Con, P=0.5993 | Day1  Scr 44±4  ICH 40±3.3  Cre 51±3.1  Day3  Con 54±4  ICH 47±4.5  Cre 53±3.3 |
| **Figure 5H**  Sham (n=10)  ICH (n=10)  Scr (n=10)  124 (n=10) | Two-way ANOVA  Row Factor  F (3, 72) =16.7, P<0.001  Column Factor  F (1, 72) = 9.31, P=0.0032 | Day1  Sham vs ICH, P<0.0001  Scr vs ICH, P=0.9791  124vs Scr, P=0.0070  Day3  Sham vs ICH, P=0.0364  Scr vs ICH, P=0.8599  124 vs Scr, P=0.0364 | Day1  Sham 52±2.906  ICH 76±3.712  Scr 78±4.899  124 61±3.480  Day3  Sham 50±2.981  ICH 64±3.266  Scr 68±3.266  124 54±3.399 |
| **Figure 6D**  Sham (n=3)  Con (n=3)  cre (n=3) | Mutiple t-test | Con VS sham  Cox2, P=0.0671  P-erk, P=0.0486  Erk, P=0.8296  p-MLKL, P=0.0090  C-cas3, P=0.1485  Fpn, P=0.0424  cre VS con  Cox2, P=0.0499  P-erk, P=0.0249  Erk, P=0.9194  p-MLKL, P=0.9293  C-cas3, P=0.0433  Fpn, P=0.0024 | Fold change (con vs sham)  Cox2, 2.734 ± 0.6822  P-erk, 1.639 ± 0.1697  Erk, 1.057 ± 0.2223  p-MLKL, 2.586 ± 0.3324  C-cas3, 2.187 ± 0.589  Fpn，1.792±0.2694  Fold change( cre vs sham)  Cox2, 5.621 ± 0.7836  P-erk,3.137 ± 0.3931  Erk, 1.087 ± 0.1693  p-MLKL, 2.541 ± 0.3365  C-cas3, 5.436 ± 0.945  Fpn，0.4219±0.00093 |
| **Figure 6E**  Sham (n=3)  Con (n=3)  Fpn (n=3) | Mutiple t-test | **Con VS sham**  Cox2, P=0.0816  P-erk, P=0.0040  Erk, P=0.3027  p-MLKL, P=0.0002  C-cas3, P=0.0168  Fpn, P=0.0077  **Fpn VS con**  Cox2, P=0.1359  P-erk, P=0.0197  Erk, P=0.3327  p-MLKL, P=0.4194  C-cas3, P=0.0433  Fpn, P=0.0041 | Fold change (con vs sham)  Cox2, 2.297 ± 0.5586  P-erk, 1.693 ± 0.0972  Erk, 1.241 ± 0.1073  p-MLKL, 2.379 ± 0.1055  C-cas3, 1.469 ± 0.0301  Fpn，2.017±0.1728  Fold change (fpn vs sham)  Cox2, 1.247± 0.07501  P-erk, 1.253 ± 0.0649  Erk, 1.107 ± 0.05738  p-MLKL, 2.185 ± 0.188  C-cas3, 1.037 ±0.04493  Fpn，3.701±0.2261 |
| **Figure 6F**  Sham (n=3)  scr (n=3)  124 (n=3) | Mutiple t-test | Scr VS sham  Cox2, P=0.043  P-erk, P=0.0071  Erk, P=0.6320  p-MLKL, P=0.0002  C-cas3, P=0.0219  Fpn, P=0.0034  124 VS Scr  Cox2, P=0.0191  P-erk, P=0.4780  Erk, P=0.9194  p-MLKL, P=0.4542  C-cas3, P=0.0274  Fpn, P=0.0444 | Fold change (scr vs sham)  Cox2, 1.378 ± 0.0411  P-erk, 2.267 ± 0.2237  Erk, 1.025 ± 0.0415  p-MLKL, 4.375 ± 0.2587  C-cas3, 1.237 ± 0.05866  Fpn，2.357±0.0605  Fold change( 124 vs sham)  Cox2, 1.031 ± 0.0816  P-erk, 1.949 ± 0.34  Erk, 1.245 ± 0.0238  p-MLKL,4.059 ± 0.2817  C-cas3, 0.965 ± 0.05452  Fpn，3.189±0.2811 |
| **Figure 6G**  Sham (n=6)  Con (n=6)  cre (n=6) | Two-way ANOVA  Row Factor  F (4, 75) = 42.86, P<0.001  Column Factor  F (2, 75) = 38.44, P<0.001 | **Con VS sham**  ATPG53, P=0.2452  Rpl8, P=0.0706  CS, P=0.9504  IREB2, P=0.0005  PTGS2, P=0.0006  **creVS con**  ATPG53, P=0.9845  Rpl8, P=0.5283  CS, P=0.0207  IREB2, P=0.0294  PTGS2, P=0.0007 | Fold change (con vs sham)  ATPG53, 0.8628 ± 0.06563  Rpl8, 0.8986 ± 0.03626  CS, 0.9925 ± 0.1068  IREB2, 2.485 ± 0.2792  PTGS2, 5.817 ± 0.9741  Fold change (cre vs sham)  ATPG53, 0.8593 ± 0.1665  Rpl8, 0.8619 ± 0.04303  CS,0.6295 ± 0.07817  IREB2,7.904 ± 2.116  PTGS2, 13.08 ± 1.136 |
| **Figure 6H**  Sham (n=6)  Con (n=6)  Fpn (n=6) | Two-way ANOVA  Row Factor  F (4, 75) = 16.06, P<0.001  Column Factor  F (2, 75) = 14.78, P<0.001 | **Con VS sham**  ATPG53, P=0.1754  Rpl8, P=0.5570  CS, P=0.9024  IREB2, P=0.0002  PTGS2, P=0.0069  **Fpn VS con**  ATPG53, P=0.3365  Rpl8, P=0.1431  CS, P=0.3961  IREB2, P=0.0106  PTGS2, P=0.03731 | Fold change (con vs sham)  ATPG53, 0.8728 ± 0.04304  Rpl8, 1.142 ± 0.1821  CS, 0.9783 ± 0.1372  IREB2, 2.39 ± 0.2216  PTGS2, 5.207 ± 1.238  Fold change (fpn vs sham)  ATPG53, 0.9673 ± 0.08312  Rpl8, 0.846 ± 0.0388  CS, 0.8356 ± 0.08427  IREB2, 1.44 ± 0.2067  PTGS2, 2.204 ± 0.1802 |
| **Figure 6I**  Sham (n=6)  Con (n=6)  124 (n=6) | Two-way ANOVA  Row Factor  F (4, 75) = 37.03, P<0.001  Column Factor  F (2, 75) = 28.93, P<0.001 | **Con VS sham**  ATPG53, P=0.4294  Rpl8, P=0.1266  CS, P=0.9136  IREB2, P<0.001  PTGS2, P<0.001  **124 VS con**  ATPG53, P=0.2039  Rpl8, P=0.2060  CS, P=0.4329  IREB2, P=0.0258  PTGS2, P=0.0025 | Fold change (con vs sham)  ATPG53, 0.9497 ± 0.05885  Rpl8, 0.77 ± 0.02602  CS, 0.9709 ± 0.2426  IREB2, 3.232 ± 0.2197  PTGS2, 4.783 ± 0.4  Fold change (124 vs sham)  ATPG53, 0.8426 ± 0.05237, Rpl8, 1.28 ± 0.3759  CS, 1.226 ± 0.1963  IREB2, 1.908 ± 0.4563  PTGS2, 2.675 ± 0.3408 |
